# Dynamical Adaptation in Photoreceptors

**DOI:** 10.1371/journal.pcbi.1003289

**Published:** 2013-11-14

**Authors:** Damon A. Clark, Raphael Benichou, Markus Meister, Rava Azeredo da Silveira

**Affiliations:** 1 Department of Physics, Ecole Normale Supérieure, Paris, France; 2 Department of Molecular and Cellular Biology and Center for Brain Science, Harvard University, Cambridge, Massachusetts, United States of America; 3 Laboratoire de Physique Statistique, Centre National de la Recherche Scientifique, Université Pierre et Marie Curie, Université Denis Diderot, Paris, France; Université Paris Descartes, Centre National de la Recherche Scientifique, France

## Abstract

Adaptation is at the heart of sensation and nowhere is it more salient than in early visual processing. Light adaptation in photoreceptors is doubly dynamical: it depends upon the temporal structure of the input and it affects the temporal structure of the response. We introduce a non-linear dynamical adaptation model of photoreceptors. It is simple enough that it can be solved exactly and simulated with ease; analytical and numerical approaches combined provide both intuition on the behavior of dynamical adaptation and quantitative results to be compared with data. Yet the model is rich enough to capture intricate phenomenology. First, we show that it reproduces the known phenomenology of light response and short-term adaptation. Second, we present new recordings and demonstrate that the model reproduces cone response with great precision. Third, we derive a number of predictions on the response of photoreceptors to sophisticated stimuli such as periodic inputs, various forms of flickering inputs, and natural inputs. In particular, we demonstrate that photoreceptors undergo rapid adaptation of response gain and time scale, over ∼ 300 ms—i. e., over the time scale of the response itself—and we confirm this prediction with data. For natural inputs, this fast adaptation can modulate the response gain more than tenfold and is hence physiologically relevant.

## Author Summary

Photoreceptors constitute the interface between the visual world and the cerebral world, as they convert light inputs into neural signals. This conversion is subject to continuous adaptation: response gain and time scale vary as a function of input history. This adaptation is ‘dynamical’ both because it depends upon the temporal structure of the stimulus and because it affects the kinetics of the response. Traditionally, theoretical studies of photoreceptors fall within one of two extreme approaches: either detailed modeling based upon the biophysics of phototransduction or functional modeling based upon phenomenological descriptions of photoreceptor response. While the former approach involves too many coupled equations and unknown parameters to allow for analytical treatments, building intuition, or predicting trends, instances of the latter approach, such as the simple linear-nonlinear model, fail to capture essential features of dynamical adaptation. Here, we develop understanding at an intermediate level. We define and discuss a phenomenological model which is simple enough to allow for full solutions and predictions, but embodies features of phototransduction well enough to capture a rich phenomenology. We demonstrate that our model reproduces data with high accuracy and can be used to make predictions on the response to sophisticated visual inputs such as natural stimuli.

## Introduction

The ability of neurons to modulate their response as a function of the environment or the task is at once a staple of neural information processing and an achievement of neural biophysics. Adaptation is at play throughout sensory systems. In peripheral sensory cells, one expects significant adaptation as these cells convert wide-ranging natural inputs into neural activity confined to a comparatively restricted range. This is true, in particular, of peripheral visual cells [1]–[11] and especially of photoreceptors [11]–[25]. A wealth of experimental data gathered over more than four decades, across species, allows the identification of universal trends in their response and adaptation properties, and renders photoreceptors an ideal testing ground for our quantitative understanding of neural adaptation.

In a typical experiment, photoreceptors are probed with flashes or steps of light, presented either in the dark or against a light background [11], [12], [14], [16], [18]–[20], [22]. In these simple protocols, ‘adaptation’ refers to the dependence of the flash or step response upon the background intensity. In photoreceptors, the response to a transient input depends strongly upon background light intensity: both the response amplitude *and* its dynamics are affected. In the dark, a photoreceptor responds to a small flash of light with a relatively large, slow hyperpolarization. Under bright background conditions, the response amplitude to the same flash decreases (reflecting a smaller gain, or ‘gain suppression’) and the dynamics of the response speed up [11], [12], [14], [16], [19], [20], [22]. The situation is further complicated in more elaborate protocols, in which the ‘background’ light intensity—thought of as a ‘*conditioning stimulus*’—itself varies in time [8], [19], [21]. Then the amplitude and dynamics of the response to a light flash or step—the ‘*probe stimulus*’—depend not only on the intensity but also upon the *time course* of the conditioning stimulus that precedes the probe stimulus. Similarly, the non-trivial dependence of the neural response on the amplitude and frequency of a periodic stimulus reflects a form of adaptation [3], [9], [17], [26]. Thus, the distinction between conditioning and probe stimuli, though useful within the contexts of some experimental protocols, may be misleading. Any given photoreceptor relies upon a single stream of absorbed photons, based upon which it produces a response at each instant in time. It is artificial—as a number of authors have noted in the past and as some of the above-referenced literature observes—to treat adaptation and response as distinct phenomena, especially if they occur on similar time scales, and a consistent model ought to address both on equal footing.

Adaptation is *dynamical* in two respects. First, photoreceptor adaptation reflects a memory of the time course of the light intensity input—we can call this ‘the dynamics of adaptation’. Second, adaptation affects the dynamics of the response itself: quite generally, gain suppression is accompanied by a speed-up of the dynamics—we can call this ‘the adaptation of dynamics’. This phenomenon is often referred to as the ‘gain-bandwidth trade-off’ [27]. Furthermore, roughly speaking, photoreceptors in the dark or in dim backgrounds tend to respond in proportion to the incident light intensity, while in bright backgrounds they respond to the time derivative of light intensity, for instance responding transiently to steps in intensity [18]. This qualitative modulation of the response as a function of background intensity supplements the quantitative effect of response speed-up. (For references on these phenomena in photoreceptors of different organisms, see Table 1 below.)

**Table 1:** A non-exhaustive list of data showing similar qualitative behavior of photoreceptors across different taxa.

Organism	Flash in the Dark: Gain Control	Flash in the Dark: Speed-Up	In Light Background: Gain Control/Weber Law	In Light Background: Speed-up	In Light Background: Bilobe Impulse Response	Frequency (Band-Passing) Response
Limulus	[72](2) [73](14,54) [74](2) [75](5)	[12](5,9) [72](2) [76](2)* [74](2) [75](1,2,3)	[12](8) [77](4) [78](2) (W = −0.7)	[12](9) [73](16)		[77](2)
Insects/Drosophila	[21](3) [4](4)	[21](3)	[21](5) [79](4) (W = −0.8) [80](11) [19](3) [81](8) [82](10) [19](7)	[21](5,6) [79](3) [80](1–8, 11) [19](8)	[82](2) [80](2,3,5,8)	[19](7(NoBP),10) [21](5,6,9) [83](7) [82](9,10) [81](2,3) [79](2) (NoBP) [80](10) (NoBP)
Turtle/Salamander/Frog (Cone)	[84](1,2) [69](3) [14](4,5) [15](7) [85](2) [86](9) [18](7,8)	[86](9) [84](1,2) [69](3) [14](4,5) [15](5)	[85](3) (W = −.7,−1.3) [87](3) (W = −1) [88](2) (W = −1) [18](11) (W = −1)	[85](3,9) [87](2,3) [89](4) [88](1) [16](1,4) [13](3,4) [15](12)	[85](2) [87](2) [88](1) [16](1,4) [13](2) [18](9)	[26](7) [89](4) (NoBP) [17](3)
Rodents/Mouse (Cone)	[90](1,2) [91](1,2)	[91](2)	[91](1)	[90](5)	[90](4)	
Primates/Humans (Cone)	[92](1) [20](1,3) [93](2)	[20](1)	[20](14) (W∼−1) [93](8) (W∼−1) [11](1)	[11](1)	[20](9)	[11](3) (NoBP)

Citation numbers are in square brackets, and the relevant figures for each follow in parentheses. In the third column of references, the specified value for W is the measured power law fit of the Weber-Fechner law. In the case of the Limulus response, data showing a distinct saddle (‘camel hump’) in the flash response are indicated by an asterisk (*). In the last column, data *not* showing band-passing characteristics are marked “NoBP.” We mention in passing that the flash response overshoot in insect photoreceptors is slight compared to that recorded in cells immediately post-synaptic to them, the laminar monopolar cells [70], [71]; we expect that a variant of the DA model applies to these laminar cells.

Any model of photoreceptor response to light intensity ought to capture this phenomenology of adaptation, namely (*i*) gain suppression, (*ii*) gain-bandwidth trade-off, and (*iii*) the transition from proportional response (to light levels) to differentiating response (to temporal derivatives of light levels) with increasing background intensity. In order to model such phenomena, it is natural to turn to the biochemical phototransduction cascade, which converts light into neural activity and which has been studied in great detail [22], [28]–[31]. Yet some of its parameters have not been measured and some of its modules, such as those involved in calcium feedback, are still a matter of investigation and, possibly, controversy (see, *e. g.*, Ref. [22]). Of greater concern is the difficulty to extract an intuitive understanding or derive qualitative predictions from the large set of coupled biochemical equations that represents phototransduction.

Here, we instead introduce a simple, phenomenological model, in the spirit of pioneering models of photoreceptor response [12], [15], [32] but differing from these in important ways. Throughout, we refer to it as the *dynamical adaptation (DA) model*. It is characterized by a dynamical non-linearity without feedback, the interplay of two time scales, and no more than a few numerical parameters. The DA model has three merits. *First*, it is simple enough to be solved *exactly*, at least formally, for *any* input. *Second*, the DA model remains rich enough to capture the phenomenology of short-term adaptation on the scale of milliseconds to seconds. Indeed, we show that it reproduces precisely a wide array of light adaptation phenomena recorded in classic experiments on turtle cone photoreceptors with flash and step inputs. *Third*, the DA model allows one to make new *qualitative predictions* on the adaptive behavior of photoreceptors, for example in response to inputs more complicated than mere flashes and steps; this is much more difficult to achieve using complicated biochemical models with many equations and a great number of numerical parameters. As an example of the predictive power of the DA model, we apply it to fluctuating inputs such as periodic or randomly flickering inputs. Such inputs are often employed in modern experiments as a means to explore a greater range of stimulus variability. It is also generally assumed that these prevent photoreceptors from undergoing significant adaptation. Contrary to this assumption, the DA model predicts that fluctuating inputs induce *fast adaptation* that depends upon both the intensity and the time course of the input. We find that the DA model reproduces the response of a salamander cone exposed to flickering light with great precision, and we indeed uncover fast adaptation in an analysis of the data. Motivated by this result, we use the DA model to make predictions about the adaptive properties of photoreceptors when these are presented with either periodic or randomly fluctuating inputs. In the case of sinusoidal light intensity, the character of the frequency-dependence depends upon the contrast of the input—an essentially non-linear effect. In the case of natural time series of light intensity, the instantaneous gain can vary more than tenfold on a fast time scale of ∼ 300 ms.

## Results

### The Dynamical Adaptation (DA) Model

We start by presenting the equations of the DA model. Subsequent sections apply these equations to various light inputs and compare the outcome to data. In formulating the DA model, we look for simple equations that capture the dynamics and adaptation of photoreceptor response. That is, we set the parameters in the DA model equations to be fixed once and for all for a given cell, so that they need not be re-fitted for different choices of conditioning and probe stimuli: any adaptive behavior is to follow entirely from the dynamics prescribed by the equations. Furthermore, we limit as much as possible the number of parameters. Saturation and adaptation effects derive from a non-linearity in the equations. Specifically, we construct this non-linearity so that it informs both gain control and temporal modulations in agreement with the ‘gain-bandwidth trade-off’: smaller gains are associated with faster responses. We present the DA model equations, then explain the intuition that lies behind them and their merit in an analytical approach.

**Figure 1:**
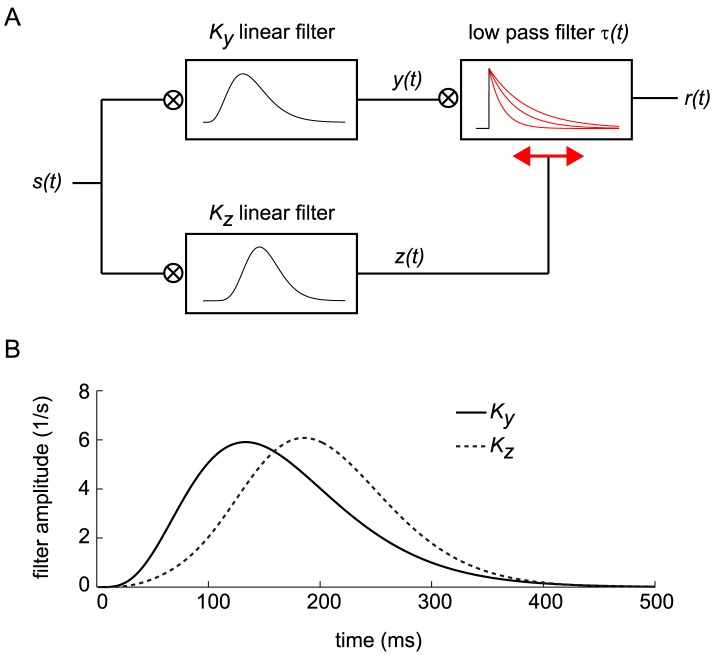
Illustration of the Dynamical Adaptation (DA) model. (A) The stimulus is convolved with two mono-lobed filters to produce the signals 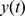 and 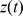. These yield the neural response according to Eq. (2). The non-linear term in the equations, which involves the signal 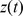, modulates gain (related to the area under the red curve) and time scale (related to the width of the red curve) in a history-dependent manner. Time scale and gain thus vary together, with small gains associated with short time scales and large gains associated with long time scales. (B) The 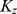 filter is broader than the 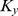 filter and, hence, can capture memory effects and mimic feedback. The parameter set is based upon the salamander data (see Methods and Table 2).

The DA model describes the photoreceptor membrane potential, 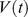, but it is more natural to write down equations in terms of the photoreceptor *response*, 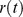, defined as the difference between the instantaneous membrane potential and the resting membrane potential in the dark:
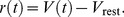
(1)The main DA model equation reads
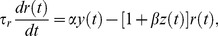
(2)where 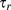, 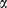, and 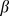 are constants. Vertebrate photoreceptors hyperpolarize in response to light, so that 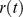 vanishes in the dark and is negative otherwise. By convention, we define 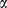 to be negative, while all other quantities are positive. The time-dependent quantities 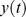 and 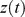 are filtered versions of the incident light intensity, 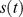, given by
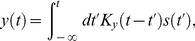
(3)

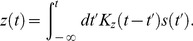
(4)The kernels 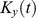 and 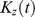 are products of monomial and exponential functions; each integrates to unity and is fully specified by a few parameters. (Explicit expressions for these functions are given in the Methods section.) The essential feature of the kernels is that they extend over comparable time scales, but with 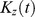 broader than 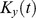 and somewhat delayed (see Fig. 1). We note that all the time scales that enter the DA model are of the same order of magnitude—several tens of milliseconds: we focus on modeling the ‘fast’ adaptation that occurs on time scales comparable to that of the photoreceptor response, and we ignore long-term adaptive phenomena which take place over seconds or even minutes [22], [25]. Equations (1–4) define the DA model.

The motivation for the form of Eq. (2) becomes apparent if we consider in turn its linear and non-linear components. If 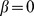, the equation is linear and the response, 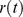, is a low-pass filtered version of the input,
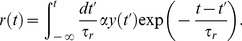
(5)Since the kernel 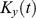 integrates to unity, in this linear version the response gain is entirely represented by the value of the parameter 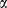. As for dynamics, the response is smoothed over the time scale of 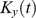 and the ‘relaxation time’ 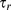. If 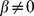, the multiplicative 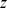-term modulates both gain and dynamics. One way of seeing this is to divide both sides of the equation by a factor 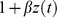, to obtain the equivalent equation,
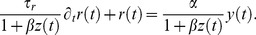
(6)The 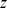-term yields effective, time-varying gain, 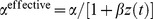, and time scale, 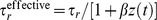. These co-vary in a manner that satisfies the gain-bandwidth trade-off: large 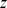 yields both small effective gain and small effective time scale, and *vice versa*. Actually, the 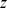-term has a more involved effect on the dynamics than the mere rescaling of the relaxation time, 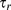, as will become clear in subsequent sections. In brief, because the effective gain is time-varying the response is in fact governed by an ‘effective kernel’ that results from a combination of the kernels 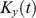 and 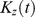; its time scale and dynamics depends upon the input's recent history.

We refer to Eq. (2) as ‘non-linear’ even though the variable 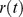 enters it only linearly; even though, in other words, the model is purely *feedforward*. The model is non-linear in that the output is *not* a linear function of the input. Throughout, when we refer to the ‘non-linearity’ in the DA model, we mean the multiplicative term, 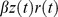, which is the only term responsible for the non-linearity of the input-output relation. A great merit of the DA model is that its feedforward form allows one to write down an *exact* solution for *any* input choice. Indeed, it is easy to verify that Eq. (2) is solved by
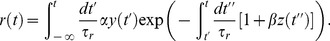
(7)One can ‘plug in’ the stimulus 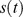 into Eqs. (3, 4, 7), analytically or numerically, and produce the model photoreceptor response. These equations provide an alternative definition of the model, and are illustrated schematically in Fig. 1. All adaptive phenomena arise because of the stimulus-dependent term that appears in the argument of the exponential and which modulates the gain and dynamics of the response. This non-linearity couples ‘conditioning’ and ‘probe’ stimuli to create a response to the stimulus history as a whole.

Our model bears similarities to various mathematical models that have been introduced in the context of molecular signal transduction and which also display interesting adaptive behaviors [33]–[38]. We return to these in the Discussion.

### The DA Model Captures the Phenomenology of Photoreceptor Response and Adaptation

**Figure 2:**
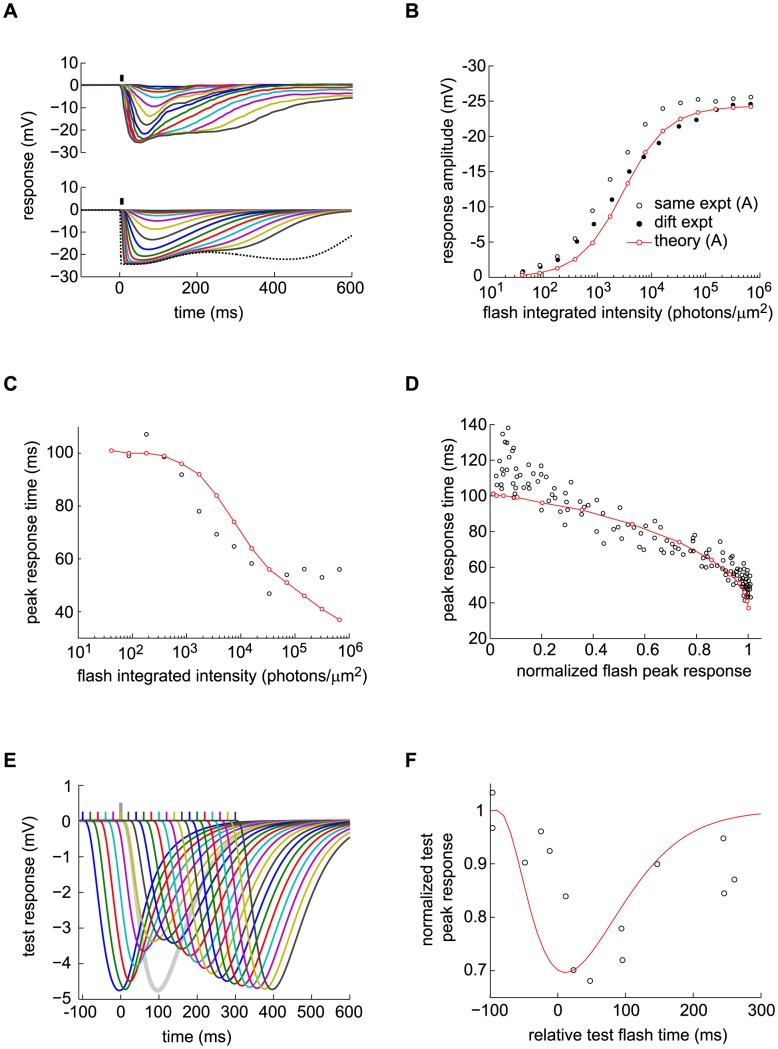
Response to single and paired flashes in the dark—comparison of data and DA model predictions. (A) *Top*: Traces of recorded hyperpolarizations in a red-sensitive turtle cone, induced by light flashes delivered in the dark. Integrated flash intensities range from 41 (lowest amplitude) to 6.7·10^5^  photons/µm^2^ (highest amplitude) in intervals of factors of 2.1. (Data from Fig. 19 of Ref. [14].) *Bottom*: DA model predictions for corresponding intensities. The dotted line represents the response to a flash 100 times more intense than the largest experimental flash. (B) Peak hyperpolarization against flash intensity for the data displayed in Fig. 2A (open circles), for a separate experiment (closed circles; data from Fig. 7 of Ref. [15]), and for the DA model predictions displayed in Fig. 2A (solid, red line). (C) Peak delay (following the input flash) against flash intensity, extracted from Fig. 2A. (Symbols are as in Fig. 2B.) (D) Parametric plot of peak delay against peak hyperpolarization (normalized by the maximum hyperpolarization). Data points (open circles) summarize several experiments (from Fig. 10 in Ref. [13]). Parameters were chosen so as to minimize the root-mean-squared error in the voltage traces, and result in faster peaks at high intensities; alternative fitting criteria could better fit the peak timing. (E) The model predicts that responses to paired flashes add non-linearly. A conditioning flash (10 ms, 560 photons/µm^2^/s) is presented at 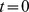 ms. A test flash of identical intensity to the conditioning flash is presented either before or after the conditioning flash, and the response is measured. The response to the conditioning flash (in the absence of any test flash) is represented as a thick, grey line, while the colored traces represent the paired flash response minus the conditioning response on its own. The test flash delivery times are indicated by small, vertical ticks of the corresponding colors. (F) Peak response to the test flash (normalized by the peak response to the conditioning flash alone) against the delay between conditioning and test flashes. Negative delays correspond to situations in which the test flash preceded the conditioning flash. Circles represent data (from Fig. 12 of Ref. [13], modified to undo a saturation correction performed there), while the solid, red line represents the DA model prediction. The DA model predictions for this figure are calculated using the parameter set BHL (see Table 2).

**Figure 3:**
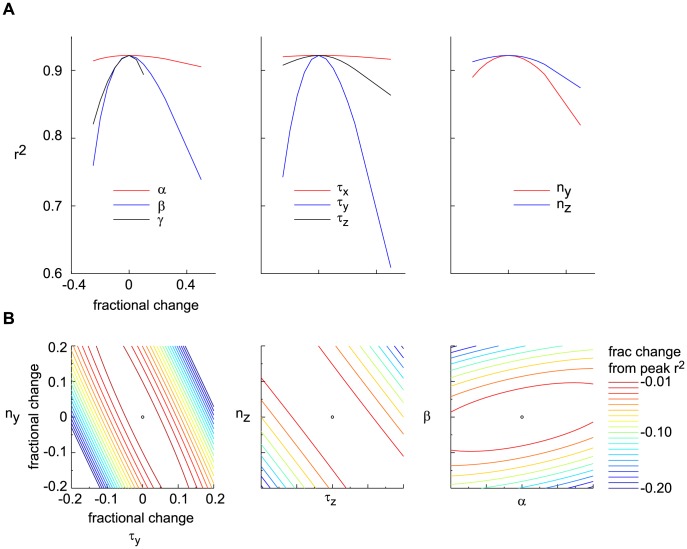
Goodness-of-fit of the model output and robustness with respect to parameter variations. (A) The three panel illustrate the relatively shallow way in which the goodness-of-fit, 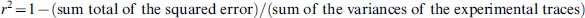, is degraded from its maximum of 0.94 by varying one of the model parameters. Optimal parameters were obtained from the traces of Fig. 2A and are given in Table 2 (BHL parameter set). (B) Contour lines representing the degradation of the goodness-of-fit with respect to variations in pairs of parameters, as a way to illustrate the reliable parameter subspace. Successive contour lines correspond to 1% increments in goodness-to-fit degradation, with the widest line corresponding to a value of 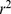 equal to 80% of its maximum. The contour lines were derived from the Hermitian matrix computed for the optimal parameter values.

**Figure 4:**
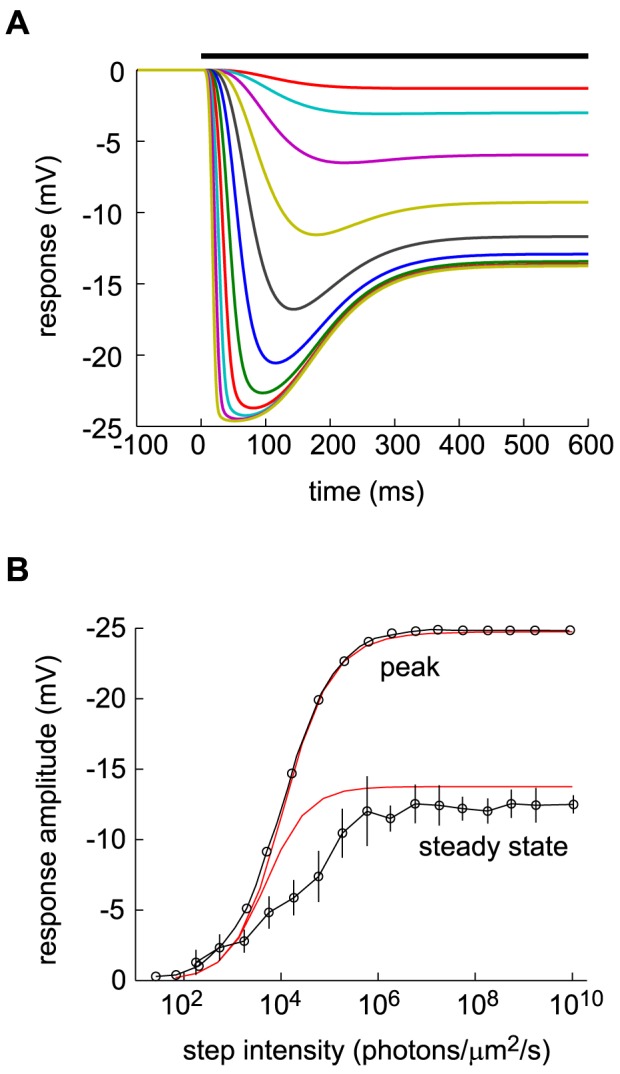
Response to light steps in the dark—comparison of data and DA model predictions. (A) DA model predictions (using the parameter set B, see Table 2). Step intensities range from 5·10^2^  to 1·10^7^  photons/µm^2^/s by factors of 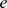. (B) Absolute value of peak hyperpolarization and steady-state hyperpolarization against step intensity. The peak response (top, black line) and the steady state response (closed circles) are from Fig. 7 of Ref. [18], and the DA model predictions (red, solid lines) use parameter set B (see Table 2). The gain was set to match the peak response.

**Figure 5:**
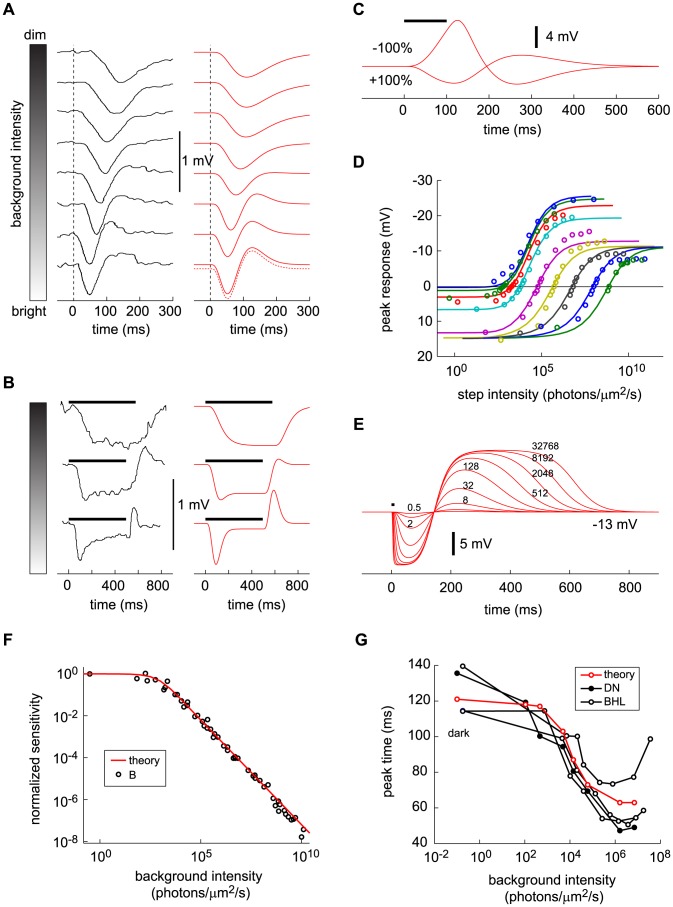
Response to flashes and steps on light backgrounds of different intensities—comparison of data and DA model predictions. (A) Experimental responses (left) and DA model predictions (right) for brief flashes of light presented at time 0 on backgrounds of increasing intensity. Data intensities and traces are extracted from Fig. 4 in Ref. [16], where the authors varied flash intensities so as to match peak response among the different background intensities. Model predictions are computed using the corresponding parameter set DN (see Table 2). The dotted curve follows Eq. 8. (B) Experimental responses (left) and DA model predictions (right) for steps of light on three backgrounds of increasing intensity. Data intensities and traces are extracted from Fig. 1 in Ref. [16]. Model predictions are computed using the corresponding parameter set DN (see Table 2). Background light intensity is indicated as in (A). (C) DA model predictions of responses to 100 ms bright (+) and dark (−) flashes equal to the background intensity, delivered at time 0. Flashes are delivered on top of a light background of 2.6·10^5^  photons/µm^2^/s (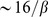, for a comparison with the strength of the non-linearity). (D) Family of peak responses to steps against step intensity. Each curve of the family corresponds to a different background of light; the background intensity increases to the right. The abscissa measures the total light intensity, so the zero crossing of each curve yields the corresponding background intensity. Negative ordinate values (upward segments of the curves) result from light steps, while positive ones (downward segments of the curves) result from dark steps. Data (open circles) are extracted from Fig. 8 in Ref. [18]. DA model predictions (solid lines) use parameter set B (see Table 2). (E) DA model predictions of responses to 10 ms flashes with large contrasts, delivered at time 0, varying from 0.5 to 2^15^ times the background intensity by factors of 4; the individual values are listed against the curves. The background light intensity, 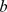, was set to 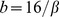. The parameter set B was used to compute the traces in both D and E (see Table 2). (F) Step response sensitivity (normalized by the response in the dark) against background intensity. Data points (open circles) are from Fig. 11 in Ref. [18]. The model prediction (red line) is computed using parameter set B (see Table 2). Both data and model satisfy the Weber-Fechner law over seven decades. (G) Delay (following the input flash) of peak responses to a fixed flash against background light intensity. Data are extracted from Fig. 5A (closed, black circles) and from Fig. 12 in Ref. [15] (open, black circles). DA model predictions use parameter set DN (red circles, see Table 2).

Classic experiments on photoreceptors have characterized their response and adaptation properties with the use of light flash and step stimuli. The resulting phenomenology is shared by different species (see Table 1). In order to assess the ability of the DA model to capture this phenomenology, we compare its output to data on one of the best-characterized photoreceptors, the turtle red-sensitive cone cell. We focus on experiments performed by three sets of researchers (Baylor, Hodgkin, and Lamb [13]–[15], Daly and Normann [16], and Burkhardt [18]—henceforth, we refer to these with the acronyms ‘BHL,’ ‘DN,’ and ‘B.’), which include five stimulus protocols: single and paired light flashes in the dark (Figs. 2 and 3), light steps in the dark (Fig. 4), bright and dark flashes against a fixed light background (Fig. 5), and bright steps against a fixed light background (Fig. 5). We emphasize that DA model parameters were fixed across *all* experiments for each of the three data sets (see Methods, Table 2). We used an optimization routine for the choice of parameters, but even parameters found by a coarse search by hand yield very similar results. In fact, it is possible to derive satisfactory curve-fitting to the three sets of data by varying only a small subset of parameters from one data set to the next. The robustness of results with respect to parameter variations is one of the strengths of the DA model.

**Table 2:** Four different parameter sets used to fit data.

Parameter	Salamander Fit	Baylor et al. (‘BHL’)	Burkhardt (‘B’)	Daly and Normann (‘DN’)
*n_y_*	4	1.5	(3)	3.7
*τ_y_* (ms)	33	38	(20)	18
n_y_*τ_y_ (ms)	132	57	(60)	67
*n_z_*	10	7	(7)	7.8
*τ_z_* (ms)	19	20	(20)	13
n_z_*τ_z_ (ms)	190	137	(140)	91
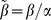 (mV^−1^)	0.16	0.044	0.067	0.074
*γ*	0.23	0.93	0.57	0.22
*τ_r_* (ms)	28	39	(50)	66
*α* (mV µm^2^ ms/photon)	a.u.	∼1.1	∼2.1	∼1.4

For data from Baylor's papers, for instance, all theoretical curves used the ‘BHL’ parameter set, with only the parameter 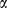 varying to match amplitudes. The parameters for each author's data set were chosen by fitting routines as described in the Methods section. The values of 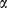 were adjusted for each experiment as simple gain adjustments (accounting potentially for light alignment, etc.), but the remaining values were all held constant. Parameter values appearing in parentheses, in column B, were not fit, but instead were set to typical values (see Methods). Traces of the response to a flash superimposed upon a light background, for each of the three parameter sets, are displayed in Fig. 10C.

#### Shape of the response: Flashes and steps of light in the dark

The most elementary behavior of the photoreceptor is its response to a flash of light in the dark: a transient hyperpolarization that returns to the resting potential over the course of 200–300 ms (Fig. 2A, top panel, data from Ref. [14]). As the flash intensity increases, the peak response grows linearly, then sub-linearly, and finally it saturates around −25 mV. The response peaks more quickly for intense flashes than for weak ones, and its tail extends further in time. Responses to intense flashes exhibit an early peak followed by a drawn-out plateau. The DA model reproduces all these features of the flash response (Fig. 2A, bottom panel). Additionally, it predicts a second peak in the response to a flash 100 times more intense than the brightest flash in the experiment of Ref. [14] (Fig. 2A, bottom panel). Such ‘camel hump’ responses were indeed recorded in other experiments [39].

We characterized the reliability of the model output by evaluating the goodness-to-fit, 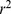; 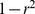 is calculated as the sum total of the squared error normalized by the sum of the variances of each trace. Our fit yields 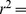
0.92. We emphasize that, while the model captures the data reliably when parameters are chosen to optimize the goodness-of-fit, the more significant observation is that the latter is not very sensitive to the precise value of the parameters. Individual parameters can be varied by a large fraction without a significant degradation of the goodness-of-fit (Fig. 3A). Furthermore, as expected the model is sensitive to combinations of parameters, so that individual parameters can be varied a great deal while maintaining a high goodness-to-fit (Fig. 3B). We conclude from these observations that the simple DA model is useful precisely because it does not require extensive fine-tuning of its parameters.

In addition to light flashes, light intensity steps are often used to characterize the behavior of photoreceptors. According to the DA model, dim steps induce a monotonic hyperpolarization while bright steps induce an overshoot: hyperpolarization peaks rapidly to a maximum value, and subsequently wanes and settles to an intermediate value (Fig. 4A). Furthermore, brighter steps induce earlier peaks (Fig. 4A), analogous to the dynamics of flash response. This scenario is indeed observed in experiments [15], [18]. In particular, the peak hyperpolarization and the steady-state hyperpolarization as a function of step intensity, as predicted by the DA model, match experimental results [18] closely (Fig. 4B).

#### Response peak amplitude and delay: Flashes and steps of light in the dark

Both the trends in peak amplitude (Fig. 2B) and peak delay (Fig. 2C), as a function of flash intensity, are captured by the DA model output. The peak amplitude exhibits the characteristic linear growth followed by saturation. The peak delay decreases with flash intensity, consistent with ‘gain-bandwidth trade-off’. Interestingly, in both the data and the model the peak delay continues to drop even after the peak amplitude has saturated (Fig. 2D).

Because of non-linearity and memory in the photoreceptor activity, the hyperpolarizing response induced by two light flashes in quick succession is *not* simply the sum of the responses to two individual flashes. If a conditioning flash is presented at time 0, the incremental response to a test flash of the same amplitude depends upon its timing relative to the conditioning flash (Fig. 2E). The ‘incremental response’ to the test flash is defined as the response to conditioning and test flashes minus the response to the conditioning flash alone. When the test flash is delivered within a ∼ 300 ms window around the presentation of the conditioning flash, the incremental response is smaller in amplitude and peaks more quickly than when the test flash is delivered much earlier or much later than the conditioning flash. Since the response to a flash is extended in time, the conditioning flash can influence the incremental response to the test flash even when it is delivered after the test flash. Baylor and Hodgkin measured the influence of the conditioning flash upon the peak amplitude of the incremental response to the test flash [13]. While the theoretical curve appears slightly shifted in time, its general shape agrees with measurements (Fig. 2F).

#### Shape of the response: Flashes and steps of light against a light background

Flashes and steps of light superimposed upon a fixed light background are popular stimuli because they can be used to assess gain and time scale modulations as a function of background intensity. Here, again, data compare well with DA model predictions. The shape of the response to a given flash (Fig. 5A) or step (Fig. 5B) delivered against a light background depends upon the background brightness. In dim backgrounds, the cone response follows the input with some delay: the response to a flash displays a single peak and the response to a step is sustained. In bright backgrounds, the cone acts more like a differentiator: the response to a flash displays an overshoot and the response to a step is transient [13], [16], [18]. In other words, cones report the light intensity itself in dim backgrounds, but something closer to its rate of change in bright backgrounds. In another context, this cross-over from low-passing behavior to band-passing behavior was explained as a way to optimize information transfer [40], [41].

The emergence of an overshoot in the flash response in bright backgrounds has a simple explanation. Essentially, it results from the delay of 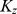 with respect to 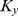: the delayed gain control suppresses the tonic response to the constant light background, which, in effect, amounts to an overshoot appended to the transient response. By contrast, in the dark there is no tonic response to suppress, and hence no overshoot can be generated. In the limit of a very bright background, calculations simplify and one can make a concise analytical statement: the DA model yields a flash response
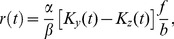
(8)where 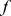 denotes the flash intensity and 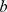 denotes the background light intensity. The factor 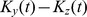 determines a universal shape of the response, while the factor 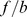 sets the response amplitude (for the derivation, see Methods). We note that in some experiments the flash response appears to slow down again under extremely bright backgrounds [13], [15]; the DA model does not account for this non-monotonic behavior.

The DA model makes additional predictions about the shape of the response to flashes against light backgrounds, which can be traced back to the simple non-linearity in the model. One prediction is that responses to light and dark flashes are asymmetric: the depolarization that follows an appreciable dark flash is larger than the hyperpolarization that follows a comparably intense light flash (Fig. 5C). The response to light and dark steps is similarly asymmetric (Fig. 5D)—an effect observed in experiments and captured by the DA model. Another prediction is that intense light flashes (many times brighter than the background) generate long-lasting response overshoots (Fig. 5E); while the duration of the first lobe in the response is fixed, that of the second lobe (the overshoot) depends upon the intensity of the flash (see Methods). Both of these effects—response asymmetry and long-lasting overshoot—result from the fact that the effective gain and time scale are controlled by the prefactor 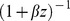 (see Eq. (6)) which behaves asymmetrically when 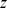 is varied above or below its set-point value, as happens when light or dark flashes are delivered against a light background.

Interestingly, asymmetrical adaptation is recorded also in human psychophysics; adaptation occurs faster following a luminance decrement than following a luminance increment [42]. This trend is observed relatively generically and is consistent with the DA model: the value of 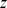 is larger immediately after a light decrement than immediately after a light increment. Consequently, the effective time scale, which is controlled by the prefactor 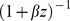, is smaller following luminance decrement than following luminance increment.

#### Response peak amplitude and delay: Flashes and steps of light against a light background

The DA model can be used to capture the behavior of the peak response as a function of both the intensity of a probe stimulus and the intensity of the light background. In the case of a step of light, the peak response depends in a complicated manner on both the step size and the background light level, and the DA model reproduces this behavior quite accurately (Fig. 5D). We note that this family of curves was obtained with a single, fixed set of parameters, without any curve-by-curve fitting.

The ‘Weber-Fechner law’, according to which the amplitude of the response to a probe stimulus depends in inverse proportion to the ‘adapting’ (background) stimulus, is observed in a broad array of psychophysical and physiological experiments. In cones, gain suppression according to the Weber-Fechner law is observed experimentally and reproduced theoretically over at least seven decades of background light intensity (Fig. 5F). Several molecular processes combine to give rise to this unified functional behavior (the Weber-Fechner law), and bleaching in particular is believed to be responsible for adaptation at high intensities. While the DA model is a phenomenological model, it reproduces the entire domain of Weber-Fechner adaptation. (For a discussion of the relation of the DA model with molecular mechanisms, see below.) We note that the form of the flash response in the case of bright backgrounds, expressed analytically in Eq. (8), exhibits precisely the Weber-Fechner form.

Response dynamics also vary with background light intensity: brighter backgrounds speed up responses. Both data and model predictions exhibit a reduction of the peak delay for increasing background intensities, followed by a characteristic saturation at high background intensities (Fig. 5G). In the DA model, this behavior comes with a simple explanation: although the ‘effective time scale’ (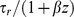) continues to drop with increasing background intensity (because 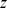 grows with background intensity), the flash response converges to a fixed shape given by 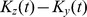, as explained above. In particular, the peak delay converges to a non-vanishing constant, the maximum of the curve 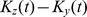.

### The DA Model Reproduces Cone Data Quantitatively and Predicts Rapid Adaptation to Flickering Input

The simple protocols we considered in the previous section make use of a transient input (the ‘probe’ stimulus) superimposed upon a constant light intensity background (the ‘conditioning’ stimulus). They provide a complete characterization of photoreceptor activity if adaptation is governed by time scales much longer than those that control the response to transient inputs. By contrast, the DA model suggests that response and adaptation occur over comparable time scales, because the quantities 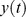 and 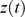 follow similar dynamics. When inputs fluctuate in time, and in particular when the fluctuations take place on time scales comparable to the photoreceptor time scales, the distinction between ‘conditioning’ and ‘probe’ stimuli fades.

**Figure 6:**
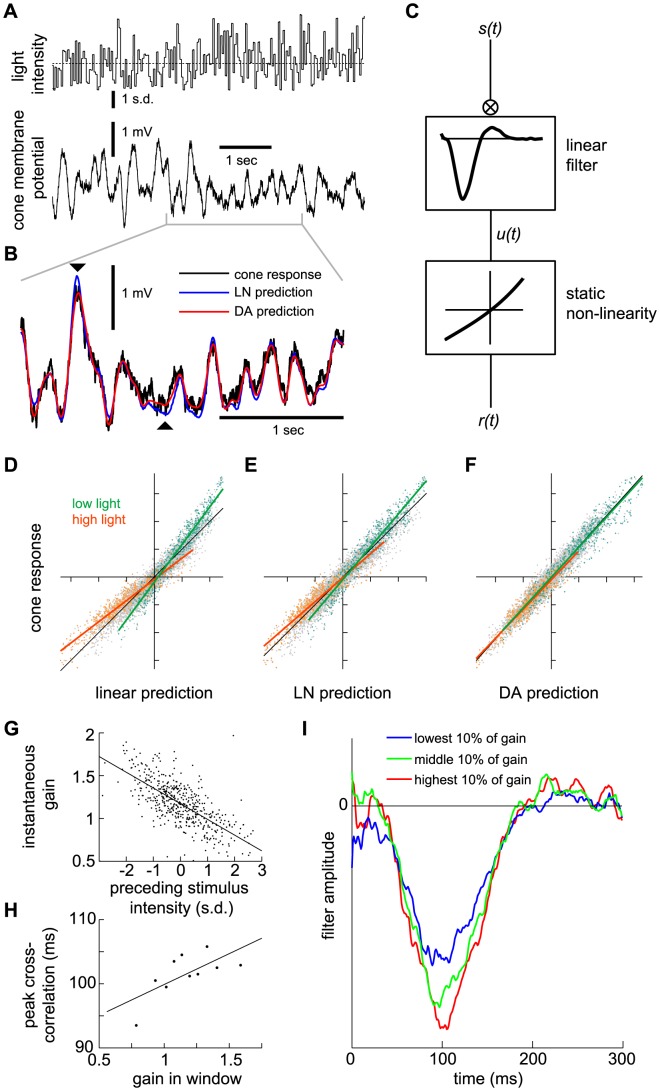
Analyses of salamander cone data with a Linear-Non-linear (LN) model and with the DA model. (A) A white-noise flickering light stimulus (top) is presented to a salamander cone while its membrane potential (bottom) is measured. (B) Enlarged cone response trace and comparison of the experimental curve, the LN model prediction, and the DA model prediction. The LN model prediction deviates from the recorded trace with a RMS residual of 0.277 (in units of response s.d., or 0.533 mV); equivalently, a 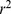 value of 0.927. The DA model has a RMS residual of 0.243 (in units of response s.d., or 0.468 mV); equivalently, a 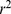 value of 0.943. The DA model follows the experimental output more closely, especially at peaks and troughs where discrepancies with the LN model are most prominent (signaled by black triangles). (C) Schematic illustration of the LN model. The stimulus is convolved with a best-fit linear filter, obtained by reverse correlation of the response to the stimulus. A static, non-linear function is then evaluated, with the output of the convolution as its argument, to produce the predicted response. (D–F) Scatter plots of the experimental cone response against the model predictions. In (D), the cone outut is compared to a linear prediction (denoted 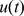 in panel C). The LN non-linearity is read off from this plot. In (E), the cone output is compared to the full LN model prediction. In (F), the cone output is compared to the DA model prediction. Low intensity (green) and high intensity (red) points are highlighted as those for which the preceding 300 ms of stimulus is in the brightest and dimmest 10%, respectively. The slope obtained from all points taken together is 1 (black line), and tick marks are 1 s. d. For the linear prediction, the slopes of the two subsets of points are 1.36 (green line, low light) and 0.76 (orange line, bright light). For the LN predictions, the slopes of the green and orange lines are 1.18 and 0.87, respectively. These differences are statistically significant (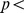
0.01, see Methods). For the DA predictions, the slopes of the green and orange lines are 0.98 and 0.94, respectively. This discrepancy is not statistically significant (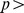
0.1, see Methods). Thus, the DA model prediction replicates the experimental output more precisely than the LN model prediction. Overall 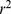 values in the three cases (D), (E), and (F) are 0.918, 0.927, and 0.943, respectively; thus, the LN non-linearity accounts for 11% of the missing variance, while the DA model accounts for 30% of the missing variance. (G) Plot of the instantaneous gain as a function of the average light intensity in the preceding 300 ms. The instantaneous gain was calculated, at each time, as the slope of the linear fit in an experimental response-versus-linear prediction scatter plot. (H) Variation of the response time scale as a function of the preceding light intensity. Instantaneous gain values along the time trace were split into ten percentile groups and, for each group, the time of maximum cross-correlation between input and experimental response was calculated (see Methods). The resulting value is plotted against the instantaneous gain value of the percentile. The slope of the best linear fit is 9.6±3.6 ms. (I) Variation of the shape and, specifically, time scale of the instantaneous best linear filter as a function of the preceding light intensity. Three linear filters, computed for the highest, middle, and lowest 10% of instantaneous gain values, are plotted. The data show that both the gain and time scale vary dynamically with light intensity.

In order to investigate adaptive properties more broadly than with flashes and steps of light, we presented cones of the salamander with a time-varying white noise, whole-field light stimulus, and we measured their responses with sharp intracellular electrodes (Fig. 6A). The DA model output closely follows the experimental membrane potential traces (Fig. 6B). As a benchmark for the DA model's performance, we compare its output to that of a model devoid of dynamical adaptation, namely the linear-non-linear (LN) model [43]–[45]. The LN model is made up of a linear filter, derived by reverse correlating the data trace with the filtered input trace, followed by a static non-linearity (Fig. 6C). Operationally, the non-linear function is extracted from a scatter plot of the linearly filtered input against output data (such as the one in Fig. 6D). The DA model trace follows data more faithfully than the LN model trace (Fig. 6B). In particular, the LN model tends to miss the peaks and troughs of the activity. This discrepancy suggests that dynamical adaptation is at play in salamander photoreceptors even under conditions of rapid light flicker: the observed instantaneous gain appears to depend upon the recent input history, whereas in the LN model any gain control is fixed as it results from a static non-linearity. In the DA model, history-dependent adaptation is embodied by the non-linear 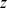-term, and its effects indeed are strongest at peaks and troughs of the response, which reflect an unusually high or low light intensity level in the recent input history.

One way to examine variable gain is to divide the data set into groups that correspond to different mean light intensities in a sliding time window of fixed duration. Here, we compared the entire data set to the 10% brightest and 10% dimmest preceding 300 ms time windows (Figs. 6D–F). The data corresponding to these two extreme regimes is not captured by a purely linear fit of the entire data set: ‘corrective gains’ have to be applied in each regime and these differ by a factor of 1.8 (Fig. 6D). Though it deviates more modestly from the data, the LN model output is not satisfactory either as corrective gains are still required to reproduce the two extreme regimes and differ by a factor of 1.4 (Fig. 6E). In contrast to the LN model's static non-linearity, the DA model accounts for moment-to-moment adaptation and captures the data without the need for corrective gains (Fig. 6F). To be precise, if corrective gains are applied in the extreme regimes, they differ by only a factor of 1.04 from each other. A statistical analysis reveals that the two corrective gains for high and low 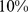 of light intensity were significantly different for the linear and LN models (both with 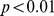), while the discrepancy was not significant in the case of the DA model (with 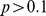, see Methods).

We emphasize that this close agreement is obtained by fitting a few numerical parameters in the DA model (see Methods), while in principle the LN model requires fitting an entire non-linear function. We also mention that the comparison, here, is with the usual formulation of the LN model, which makes use of a single temporal filter. Generalizations of the LN model that make use of more than one temporal filter (see, e.g., [46]) would naturally achieve a higher performance. However, in the absence of a general prescription on how to combine the various filters, an LN model with, e.g., two temporal filters would require fitting an entire surface (rather than a line) to the data. The DA model actually suggests a specific prescription for the case of photoreceptors; namely, that the second, slower temporal filter should act as a divisive modulation of the first, faster temporal filter. Indeed, if 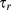 is small with respect to intrinsic times scales of the input or in general at high background light levels, the DA model reduces to a two-filter LN model in which the non-linearity is a simple divisive one (see, e.g., Eq. (15) below).

The temporal filters used in the DA model indicate that dynamical adaptation occurs over a time scale of ∼200 − 300 ms, and indeed we obtain a clear negative correlation between the ‘instantaneous gain’ of the salamander cone response and the mean light intensity over the preceding 300 ms (Fig. 6G, see the figure caption and Methods for a definition of the instantaneous gain). These data also suggest that the response time scale varies in a correlative manner with the instantaneous gain (Fig. 6H): the response of the salamander becomes faster at moments following periods of high light intensity (Fig. 6I, see also Methods). As before, a careful analysis reveals that this trend is statistically significant (with 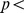
0.05, see Methods).

### The DA Model Predicts Different Frequency Dependences for Different Input Contrasts

**Figure 7:**
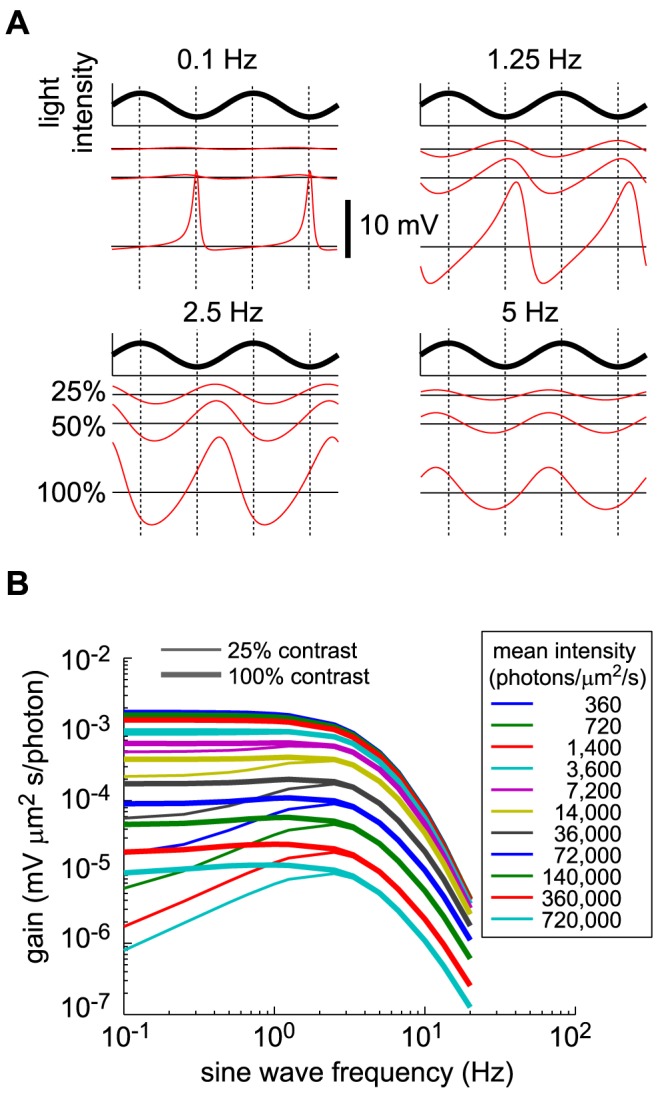
Response to periodic inputs—DA model predictions. Parameter set B was used for theory curves in this figure (see Table 2). (A) Traces of model responses (thin lines) to 25%, 50%, and 100% contrast sinusoidal inputs with frequencies 0.1, 1.25, 2.5, and 5 Hz, superimposed on a background light intensity of 3.6·10^5^  photons/µm^2^/s. The thick line represents the input. The abscissae are scaled so as to allow for two periods. Horizontal lines for each trace represent the potential when the input sinusoid has 0 amplitude. (B) ‘Effective gain’, calculated as the ratio of the trough-to-peak amplitude of the response to the through-to-peak amplitude of the input. Different colors correspond to different background intensities, varying from 360 to 7.2·10^5^  by factors of ∼2. At low input contrast (thin lines), the DA model behaves as a band-pass filter, while at high input contrast (thick lines) it behaves nearly as a low-pass filter.

Periodic stimuli represent a standard choice for probing the temporal aspects of a response function, and we use them here to illustrate the temporal properties of adaptation in the DA model. We present the model photoreceptor with a sinusoidal fluctuation superimposed upon a constant light intensity background. Here, we define the ‘stimulus contrast’ as the fractional maximum deviation from the mean light intensity. The DA model predicts that the frequency-dependence of the response itself depends upon stimulus contrast. Low-contrast inputs induce a linear, phase-shifted response at all frequencies (Fig. 7A). High-contrast inputs generate qualitatively different output traces, with shapes that depend upon frequency. At intermediate frequencies (1.25 Hz and 2.5 Hz in Fig. 7A), the skewed output traces predicted by the DA model are reminiscent of measurements in the primate outer retina, for which a model akin to the DA model has been advanced [9]. The data presented by Lee et al. [9] corresponds to horizontal cell recordings, the retinal neurons postsynaptic to photoreceptors; while it reflects adaptive processing in photoreceptors, it also includes further steps of processing in photoreceptor terminals and horizontal cells. For this reason, we have not attempted a direct quantitative comparison with the output of the DA model. At low frequencies (0.1 Hz in Fig. 7A), the response follows the input closely, without appreciable delay, with only near-instantaneous gain modulation at play.

The response plots discussed above yield an interesting prediction on gain control. When we consider the *frequency-dependent gain* — the ratio of the trough-to-peak amplitude of the (periodic) response to the through-to-peak amplitude of the periodic input — two qualitatively different behaviors emerge in bright backgrounds (Fig. 7B). As expected from flash responses, for low-contrast input the gain is suppressed at low frequencies and has a maximum at a given frequency that reflects the time scales in the DA model, provided that the background light intensity is appreciable. (In fact, if 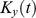 and 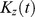 are defined to each integrate to unity, as we have it here, the gain vanishes in the limit of low frequency.) Such band-passing behavior was observed in turtle cone [17] and salamander cone [26] experiments. For high-contrast inputs, the gain remains appreciable at low frequencies. When the model photoreceptor is exposed to high-contrast, slow, periodic input, its response simply follows the input with saturation; in the limiting case of a very intense light background, the response oscillates between zero and its saturation value, 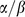. There remains a small gain suppression at low frequencies due to the non-linear, saturation property of the photoreceptor response. Thus, when exposed to high-contrast inputs the model photoreceptor responds in a ‘low-passing’ manner. While we expect experiments to confirm this behavior, quantitative comparisons remain to be carried out.

We note that the frequency dependence of the gain in the high-contrast case obtained from the DA model is very similar to the experimental frequency dependence presented in Fig. 1A of Ref. [3]. As in the case of Ref. [9], Ref. [3] reports on recordings of horizontal cells submitted to periodic light input. It proposes a model that makes use of a feedback, frequency-dependent, divisive non-linearity. The DA model offers an alternative explanation.

### The DA Model Predicts Large Modulation of Gain by Natural Flickering Inputs

Flicker stimuli are used quite commonly to measure receptive fields (see, e.g., [45]). These can change as a result of adaptation (to different levels of light intensity, contrast, or other stimulus parameters), and studies of adaptation often use flicker stimuli to evaluate the receptive field under different conditions (see, e.g., [1], [6]–[8], [47]). Thus, the flicker stimulus is meant as a ‘probe’ to test performance of the system in different ‘states’. Here, we show that this stimulus *itself* induces substantial adaptation in the system, so the system actually experiences a range of states even during the probe stimulus. The issue is not a purely academic one, since in many natural situations individual photoreceptors indeed receive a flickering input. The time scales in natural situations are a function of spatial modulations and motion in the visual scene, as well as observer and eye movements. In humans, saccades occur every 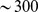 ms [48], thus producing flicker at individual photoreceptors with a time scale of the order of response time scales. Furthermore, in a natural visual scene, light intensity varies in space by about four orders of magnitude [48], yielding large-amplitude flicker from eye movement.

**Figure 8:**
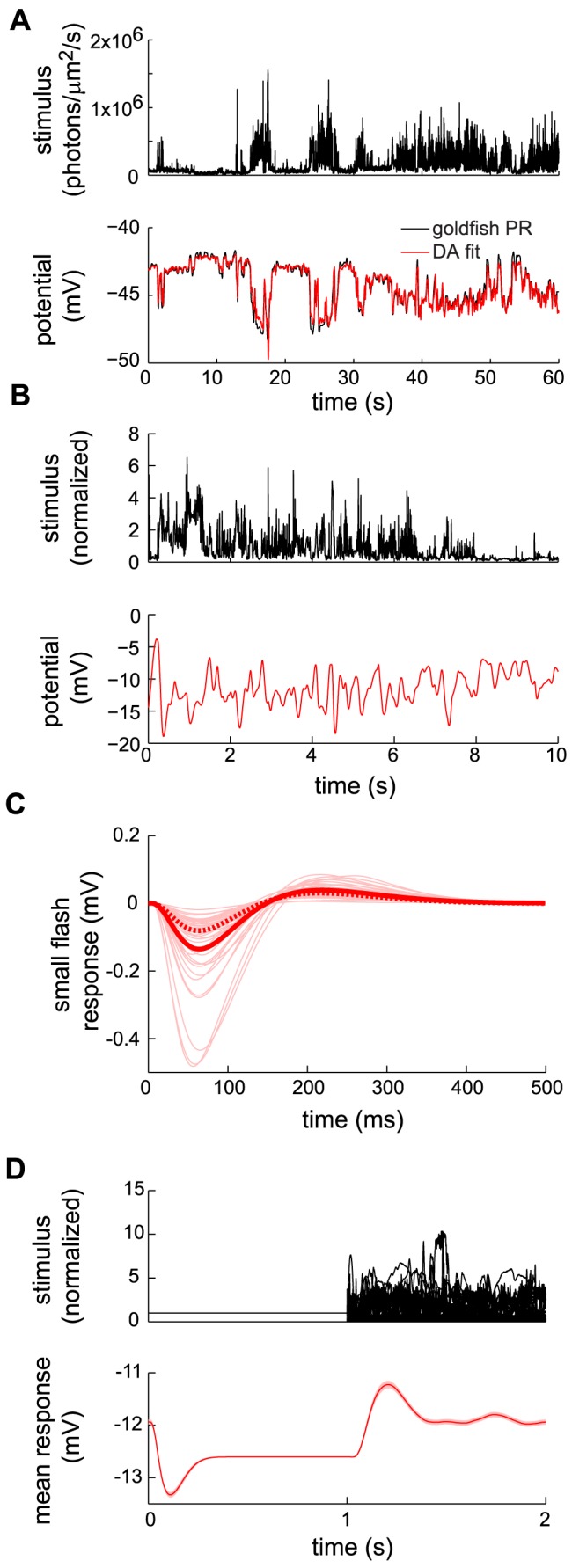
Response and dynamical adaptation with respect to natural fluctuating inputs—DA model fit and predictions. (A) *Top*: Sample of a light intensity trace, from the natural time series in Ref. [50]. *Bottom*: The corresponding DA model response trace (red), superimposed upon goldfish cone recording (black) from Ref. [49]. The agreement between the two traces is quantified by an 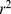 values of 0.934. The parameters 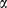, 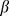, and 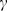 were fitted to the data; otherwise parameters from set B were used (see Table 2).(B) *Top*: Different clip from the same light intensity trace as in (A), from the natural time series in Ref. [50]. *Bottom*: The corresponding model response trace. (C) DA model predictions of responses to small (100-photon) flashes superimposed on the fluctuating natural light intensity. The flash is presented at time 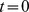. Thin pink curves represent individual flash responses, while the thick red curve is the average over all such responses. The weakest (1st percentile) and strongest (99th percentile) peak responses are measured as −0.0238 mV and −0.5524 mV, respectively, *i. e.*, they differ by a factor greater than 20. The dotted thick red curve is the flash response in the presence of a constant background matched to the mean of the fluctuating input. The dotted red curve peaks at 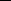
0.081 mV, while the solid red curve peaks at 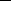
0.135 mV. Thus, individual flash responses vary greatly as a function of background history, and their mean is offset with respect to the constant-background case. (D) Response to a fluctuating input with time-varying contrast. *Top*: Superimposition of several input traces. The standard deviation of the flicker switches, suddenly and periodically, from its natural value to zero, with a period of 2 s, while its mean remains constant. *Bottom*: Trace of the mean DA model response to the time-varying flicker. (The thick red line represents an average over multiple natural stimuli. The pink area represents the standard error of this average.) Each switch is signaled by an over- or under-shoot in the mean response, depending upon the direction of the switch. The ‘steady-state’ mean response is greater (more hyperpolarized) in the constant-background half-period than in the fluctuating-background half-period. Parameter set B was used for all theory curves in (B), (C), and (D) (see Table 2).

Endeman and Kamermans [49] recorded from a goldfish cone which was presented with a clip of the naturalistic light intensity time series measured by van Hateren [50] (Fig. 8A, top panel). We digitized the goldfish cone voltage trace and fitted the DA model to it (Fig. 8A, bottom panel, see also Methods). For this specific movie clip, the gain in the goldfish cone response varies in time by a factor of three; the quantitative agreement (with 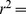
0.934) between the experimental and theoretical traces demonstrates that the DA model replicates the modulation in the reponse properties also in the case of natural inputs, in which fluctuations can be more severe than in laboratory conditions.

In order to explore the properties of the DA model in response to natural stimuli further, we calculated its response to a different clip of the same naturalistic stimulus (Fig. 8B, top panel), deeper in the non-linear regime (see Methods for details on model parameters). The series varies over close to three orders of magnitude on scales ranging from tens of milliseconds to seconds. The DA model response to this input (Fig. 8B, bottom panel) exhibits overall gain compression: the output varies over less than a single order of magnitude. But how can we extract the rapid, moment-to-moment adaptation induced by the fluctuating input? An intuitive way to uncover rapid adaptation is to superimpose a set of dim flashes upon the natural light intensity series. The ‘impulse response’ (*i. e.*, the response to the complete input *minus* the response to the natural time series alone) reveals the moment-to-moment adaptation that occurs in the model photoreceptor: its amplitude varies as a function of time. It can be either smaller or larger than the response (Fig. 8C) to an identical flash superimposed upon a fixed light background matched to the mean intensity in the natural series. Furthermore, moment-to-moment adaptation is significant: impulse response amplitudes vary by more than twentyfold (Fig. 8C).

From the non-linear structure of the DA model it further follows that the mean impulse response has a greater amplitude than the impulse response in the case of a matched constant light background (Fig. 8C). In other words, on average the model photoreceptor is more sensitive in a fluctuating visual environment than in a static one. The variations of impulse response amplitude follow from the fact that the instantaneous gain depends upon light received during the preceding 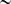
300 ms. And the enhanced average impulse response follows from the model's property that, in a bright visual environment, moments of brighter light only suppress the gain by a little bit while moments of dimmer light boost the gain appreciably.

Yet another manifestation of dynamical adaptation as captured by the DA model lies in the difference between the average response to a flickering input and the response to a constant light input with matched mean. In order to examine this effect, we constructed an input in which 1 s windows of natural intensity time series alternated with 1 s windows of constant light with matched mean (Fig. 8D, top panel). We calculated the model response over instantiations of natural time series, and derived two conclusions from the average response trace (Fig. 8D, bottom panel). First, on average a transient hyperpolarization follows the onset of the constant light input, while a transient depolarization follows the onset of flicker. Second, the ‘steady-state’ average response to flicker is depolarized as compared to the steady-state response to constant light. The transient hyperpolarizing and depolarizing responses arise because a light-adapted photoreceptor is more sensitive to negative (*i. e.*, hyperpolarizing) deflections in the input than to positive (*i. e.*, depolarizing) ones (Fig. 5D). This asymmetry biases the average steady-state response to flickering input toward depolarization, as compared to the response to constant, mean-matched light.

The above arguments appear to be quite general, and should apply to cases in which flickering inputs are drawn from distributions other than the van Hateren series used here. In Methods, we discuss the case of Gaussian flickering inputs, often used in experiments. For that choice of inputs, we can work out analytical results, which confirm the above arguments and agree with numerics. Furthermore, the analytical results illustrate the fact that adaptive effects depend not only upon that magnitude of the flicker but also upon its temporal structure (see Methods).

### Putative Connection of the DA Model to the Biochemistry of the Phototransduction Cascade

The DA model is a phenomenological model that makes no explicit reference to the mechanisms of phototransduction. A number of studies [22]–[24], [28]–[31] have examined the mechanism by which light is converted into electrical activity in photoreceptors. They have revealed the beautiful intricacies of the biophysics of phototransduction at the molecular level, but the resulting set of equations is too complicated to be used, as a whole, for developing intuition or making qualitative predictions. Phenomenological and mechanistic approaches are complementary in the purpose they serve; nonetheless it is worthwhile to look for possible connections.

**Figure 9:**
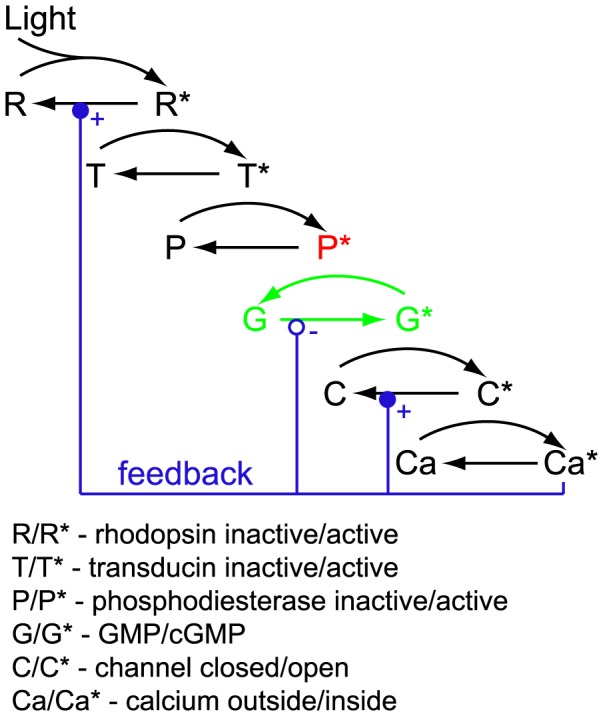
Schematic illustration of the phototransduction cascade. Initially, photons are absorbed by rhodopsin molecules. This triggers a sequence of biochemical reactions resulting in photoreceptor hyperpolarization and calcium influx. We highlight the reaction step in which activated phosphodiesterase (red) increases the rate of conversion of cyclic GMP to GMP (green). The documented calcium feedback mechanisms (blue) include positive and negative regulations of reaction rates (denoted by the symbols + and − respectively).

The hyperpolarizing response of a photoreceptor to light results from the closing of channels, due to the transformation of cyclic GMP (cGMP) into GMP through the action of activated phosphodiesterase. The molecular steps of the phototransduction cascade are illustrated schematically in Fig. 9. In order to explain the properties of cone response at the molecular level, it is necessary to understand the nature and relative relevance of the non-linearities at each stage of the feedforward cascade. Because the reduction of the cGMP concentration, 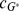, depends upon the concentration of activated phosphodiesterase, 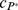, *and* upon its own concentration—the concentration of *cyclic* GMP (*i. e.*, the *activated* form of the substrate)—the corresponding phototransduction step is non-linear even at relatively low light intensities (see Eq. (9) below). This reaction is often presented as the dominant source of non-linearity in the cascade (see, *e. g.*, Refs. [23], [25] and references therein). At high light levels, other sources of non-linearity may come into play. Pigment bleaching becomes relevant over the three or four upper decades of illumination, up to 10^10^  photons/µm^2^/s [18]. That is, in this range of illumination the very first reaction in the cascade, between photons and rhodopsin, becomes non-linear due to the limited pool of rhodopsin molecules. Whether similar substrate-limited non-linearities occur at intermediate light levels in the case of transducin and phosphodiesterase is as yet unclear for cones. (While there are indications that the transducin and phosphodiesterase steps in the cascade may be substrate-limited in rods [51], experiments on cones suggest that phosphodiesterase does not become limiting until the photopigment is already completely bleached [52]. These experiments, though, were performed on membrane preparations from cones and hence do not take into account morphological effects of natural phototransduction. Cone morphology may have a significant influence on the activation of phosphodiesterase by diffusing activated transducin molecules.)

It thus seems that the site of adaptation moves from the back end of the feedforward cascade—namely, the GMP step—to the front end at very high light intensities. (In an interesting analogy, within the retina as a whole, the main site of light adaptation also moves from downstream processing—namely, the transfer from bipolar to ganglion cells—to the front end—namely, cones—at high light intensities [11].) Here, we focus upon the GMP step of the phototransduction cascade, as its non-linearity appears to play a dominant role over a major part of the natural range of light intensities. The analysis of earlier non-linearities would be similar, as their mathematical form is similar. The inactivation of GMP by phosphodiesterase can be modeled as
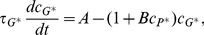
(9)where 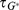, 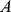, and 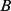 are constants. The photoreceptor response grows in proportion with the deviation from the resting value of the cGMP concentration,
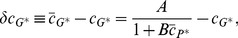
(10)where 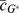 and 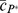 are the resting values of the concentrations of cGMP and phosphodiesterase, respectively. Inserting Eq. (10) into Eq. (9), we obtain an equation for 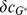, as
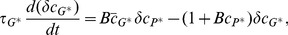
(11)where 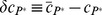 is the deviation of the phosphodiesterase concentration from its resting value. Equation (11) is similar to the central equation of the DA model, Eq. (2): here 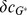 plays the role of the response, 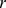, and 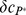 is an intermediate, light-responsive quantity analogous to 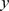 and 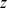 in the DA model.

The essential difference between Eqs. (2) and (11) is that, in the former, the quantities 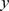 and 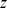 vary on different time scales, whereas in the latter, the two phosphodiesterase-related quantities vary on the same time scale. In the DA model, the action of 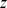 is somewhat slower and slightly delayed with respect to that of 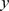. Indeed, 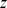 can be written as resulting from a convolution with the sum of two kernels, one corresponding to the dynamics of 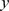 and the other to slower dynamics (see Eq. (13) in Methods). The fast component of 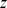, which operates on the time scale of the response, i.e., the time scale of 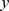, can be identified with the non-linearity inherent in the feedforward pathway of the phototransduction cascade, discussed above. The slow component of 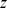 can be interpreted as mimicking the delayed effects of feedback loops in phototransduction, i.e., biophysical reactions that occur beyond the main cascade (discussed above). The quality of our fits to data suggests that, at least within the experimantal range we considered, the complicated feedback processes involved in phototransduction may be well approximated by a simple feedforward non-linearity.

## Discussion

### Summary

We introduced a new phenomenological model that captures the response and adaptation properties of cone photoreceptors. The DA model is expressed as a first-order differential equation in time (Eq. (2)) and relies upon a single non-linearity. Because of the interplay of a few time scales, response properties depend upon recent history. Both response gain and dynamics are influenced by the input history. Thus, the DA model provides an example of truly dynamical adaptation. The simplicity of the model allows for an exact analytical solution for any input time course (Eq. (7)) and for straightforward numerical calculations.

We evaluated DA model outputs for inputs that have been used historically to characterize adaptation, namely flashes and steps of light, and we found that the DA model captures the phenomenology of adaptation qualitatively, and in most cases also quantitatively. Specifically, it reproduces gain compression and dynamical modulation of the response to large ‘probe’ stimuli (flashes and steps) (Figs. 2 and 4), as well as gain control and dynamical modulation as a function of ‘conditioning’ stimuli (Fig. 5). What is more, we found that the transition from a monophasic flash response in dim backgrounds to a biphasic flash response in bright backgrounds emerges naturally from the DA model (Fig. 5). Interestingly, also, while we fitted the model to data that did not present these, it predicted double-peaked (‘camel hump’) responses to intense flashes (Fig. 2A, bottom panel); responses of this characted indeed have been recorded experimentally [39].

When we stimulated the DA model with randomly flickering inputs, we found that it can reproduce salamander cone data with great precision (Fig. 6). In particular, it corrects systematic errors that appear if the dynamical character of adaptation is ignored (as in LN models). Furthermore, the DA model predicts fast, moment-to-moment adaptation, controlled by a time scale of about 300 ms, even in the presence of rapid flicker. A careful analysis of salamander cone data indeed uncovered this form of fast adaptation (Fig. 6).

The fundamentally dynamical nature of adaptation in the DA model implies other non-trivial response behaviors when the model photoreceptor is exposed to fluctuating inputs, such as periodic inputs or flickering inputs. In the case of periodic inputs, it predicts a qualitative change of the frequency-dependence of the response when contrast in varied: At low contrast slow inputs are suppressed, while at high contrast slow inputs elicit maximum gain (Fig. 7).

In the case of randomly flickering inputs, the gain in response to transient stimulation varies significantly on a fast time scale (Fig. 8). Furthermore, the mean photoreceptor output itself is modulated by the amplitude of fluctuations (Fig. 8). Such a coupling between the mean and fluctuations about the mean is a signature of non-linearity.

The DA model is a worthwhile compact description of phototransduction, especially as several of the important numerical parameters involved in the molecular cascade have not been measured, the forms of some of the non-linearities have not been determined, and the feedback mechanisms—in particular the multiple calcium feedback mechanisms—are still a matter of investigation (see, *e. g.*, Ref. [22]). We have given a heuristic interpretation of the DA model in terms of phototransduction biochemistry. In the light of this interpretation, adaptation is seen as the result of a fast process inherent to the *feedforward* branch of phototransduction, supplemented by a slower, presumably feedback, process still accurately mimicked in the DA model by an additional feedforward term. Here, ‘feedback’ refers to a process in which the output state of the photoreceptor would affect an ‘upstream’ biophysical interaction. But this does not mean that the DA model provides a complete description of (feedback) adaptation. In the feedforward DA model, gain and time scales co-vary. Some calcium-related processes in feedback adaptation may work differently. Experiments indicate that calcium concentration can modulate response gain while leaving time scales unchanged [53]. Furthermore, calcium dynamics seem to involve much longer time scales than those of concern here [25], [54], [55].

### What Is (Dynamical) Adaptation?

Historically, light adaptation was defined with experiments that used a ‘conditioning’ stimulus and a ‘probe’ stimulus. The neuron under study was exposed to a conditioning stimulus for some time, and then its response to a probe stimulus was measured; adaptation was defined in terms of the difference between the responses to the probe stimulus with and without conditioning stimulus. Typically, conditioning stimuli were chosen to vary slowly in time or not vary at all, as in the case of a constant light intensity background, and probe stimuli were devised as transient variations in light intensity, such as flashes or steps.

Quite generally, neural activity saturates in response to large stimuli. One concern, in defining adaptation, was to disambiguate this simple gain compression from a more involved effect of the conditioning stimulus [13], [14]. Clearly, for this one needed a model of the gain compression. The LN model was often used as such a model: instantaneous gain compression was ascribed to the shape of the non-linear transfer function (the ‘N’ part of the LN model), while ‘true adaptation’ was inferred from conditioning stimulus-dependent changes in the amplitude and shape of the linear filter (the ‘L’ part in the LN model) [6], [8], [46].

Thus, light adaptation is often defined in a model-dependent manner that may lead to some amount of confusion. For example, if a system is invested with dynamical non-linearities—as is generally the case for neural systems—it is unnatural to disambiguate ‘gain compression’ and ‘true adaptation’ with the use of a static non-linearity. But even if one ignores this caveat, the definition of adaptation in terms of ‘conditioning’ and ‘probe’ stimuli may be problematic. The definition is suitable if the time scales of response and adaptation are very far apart. Then any stimulus can be divided into a slowly varying component, which ‘conditions’ the system, and a rapidly varying component, with which the system is ‘probed’. But if the time scales of response and adaptation are comparable, as is the case for photoreceptors, then the distinction between ‘conditioning’ and ‘probe’ stimuli becomes artificial. This is especially true when the input itself varies over these time scales. Put differently, photoreceptors adapt and respond concomitantly.

In experiments in which the response properties of a cell are modulated by the intensity of the input fluctuations, rather than by changes in its mean, it is customary to invoke ‘contrast adaptation’. In our case, too, one can say that the photoreceptor undergoes a kind of contrast adaptation, as its sensitivity is modulated by the intensity of input fluctuation (see Fig. 8). But this terminology may be misleading because, again, all three time scales—that of response, that of adaptation, and that of flicker—are comparable. For this reason, we prefer to talk about dynamical adaptation. In a model such as the DA model, and in reality, adaptation is dynamical in at least two ways. There is ‘the dynamics of adaptation’: the way in which response properties adjust depends upon the structure of the history of the stimulus, not only upon a single number. There is also ‘the adaptation of dynamics’: not only does the gain change as a function of the experimental conditions, but the response kinetics also vary.

Regardless of the specific form it takes, adaptation is often viewed as a change of model parameters—gains or time scales, for example. But a complete model should incorporate the apparent change of parameters, on several nested time scales, as a natural result of its (possibly very complicated) dynamics. A number of studies have addressed this issue, and in particular have proposed models with temporal properties that vary adaptively [6], [10], [46], [47], [56]–[59]. Similarly, the simple DA model can account for the phenomenology that can appear as a change of LN model parameters, namely fast adaptation over a few hundred milliseconds. We have argued that, in a case such as this, response and adaptation are inseparably intertwined concepts. In the case of longer-term adaptive phenomena (for example, those that result from photopigment regeneration), one can invoke slow parameter changes in the DA model. Here, ‘adaptation’ can be defined more easily. But, again, ultimately one would like to construct a richer model that incorporates dynamics over the longer time scales of interest. In this upgraded description, there will be no formal distinction between ‘adaptation’ and ‘response dynamics’. In this sense, ‘adaptation’ is an elusive notion: once understood in terms of a system’s dynamics, it no longer stands as an independent feature [10], [57]. Instead of speaking of adaptation, it may be more natural to characterize a neural system or a set of response phenomena by the time scales and non-linearities that govern the dynamics. In the case of the DA model, photoreceptors are described by the interplay of three time scales and a single, multiplicative non-linearity according to which gain and dynamics are modulated by a signal, 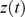.

### Phenomenological Nature of the DA Model

The DA model is a phenomenological (or functional) model. It came about as we were searching for a simple model that could capture data, and in particular the dynamical aspects of adaptation, quantitatvely. Somewhat to our surprise, we found that it reproduced a large quantity of observations collected over the past four decades. It also corrected systematic errors made by LN models—which, incidentally, fail to describe the dynamical aspects of adaptation—when it was checked against photoreceptor reponse traces that we recorded. Now, the photoreceptor is likely the best understood neural cell and the biochemistry of phototransduction is identified in some detail; it can be modeled as a relatively large set of coupled non-linear equations. A question, then, follows: Have simple, phenomenological models, such as the LN model or the DA model, any reason of being? We believe that the answer is affirmative, and we explain here why.

Biochemical models involve not only many coupled non-linear equations, but also a large set of numerical parameters, many of which cannot be measured directly. Thus, it is very difficult to explore the parameter space of these models and to extract from them generic behaviors and testable predictions. By contrast, a phenomenological model of a cell’s response can be tractable enough that generic behaviors and robust predictions be established. Phenomenological models are thus useful to identify ‘computational modules’, which can be sought after in more complicated mechanistic models. For example, the DA model displays the computational power of the interplay of two time scales through a feedforward non-linearity.

Phenomenological (or functional) models have proved fruitful in neuroscience. Besides the reasons just mentioned, this is because they embody what a post-synaptic neuron or, more generally, a neural circuit ‘cares about’. While the study of the intricacies of the phototransduction cascade is eminently interesting, ultimately the input-output relation of photoreceptors is relevant to downstream visual processing, irrespective of biochemical details. Moreover, phenomenological models are useful in establishing connections between systems that share functional commonalities but may differ greatly in their mechanistic aspects. For example, the DA model is akin mathematically to models of signaling in non-neural cells [33]–[38]. Phenomenological models also come with a generalization power: they can be modified to describe other systems. We indeed expect that variants of the DA model will be useful to study the computational properties of visual or sensory cells other than photoreceptors, which do not rely upon any kind of phototransduction but which do display a similar phenomenology in their input-output relations. Finally, phenomenological models can be of use in analyzing mechanistic models. Here, we have used insights gleaned from the DA model—namely, that adaptation may result from feedforward coupling and that a simple non-linearity involving two time scales may be responsible for it—to examine the phototransduction cascade and to suggest a putative key step in the latter.

### How Is the DA Model Different from Earlier Phenomenological Models?

The DA model is similar in spirit to the pioneering phenomenological models of photoreceptors by Fuortes, Hodgkin, Baylor, and Lamb [12], [15] and by Carpenter and Grossberg [32]. The oldest model, put forth by Fuortes and Hodgkin [12], is made up of a succession of linear filters followed by a feedback non-linearity. The cascade of filters in their model plays the role of the filter 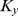 in the DA model, and the non-linearity governs adaptive phenomena as in the DA model. In another class of models, advanced later by Baylor, Hodgkin, and Lamb [15], again a succession of linear filters controls the variation of an intermediate quantity (presumed to be the concentration of some chemical) which then translates into the membrane potential of the cell. But this quantity induces its own removal, through a feedback process on decay rates.

These earlier phenomenological models and the DA model are similar in that they rely upon an initial linear filtering of the input and a subsequent non-linear transformation. The major difference between the two, however, is that earlier models come with a *feedback* non-linearity while the DA model comes with a *feedforward* non-linearity. The former involves higher powers of the model output, so that a linear analysis (such as fitting an LN model) would reveal *output-dependent* effective parameters. By contrast, the latter remains linear in the output; as a result, in a linear analysis effective parameters are *independent of the output* state of the system. Roughly speaking, in a feedback system the output can affect the earlier stages, while in a feedforward system it does not—only the input does. In the Fuortes-Hodgkin model [12], for example, the role of our delayed signal, 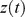, is played by the cell output which enters the equation non-linearly; thus, adaptive properties depend upon the value of the output, whereas in the DA model they are affected only by the value of the input. Biochemical feedback loops in phototransduction are well-documented—so is a feedforward model bound to be useless? There are at least two reasons for which a feedforward model applies well to this system. First, adaptation may be carried out in both the (complicated) feedforward part of the phototransduction cascade and in its feedback loops simultaneously (see section ‘Putative Connection of the DA Model to the Biochemistry of the Phototransduction Cascade’ above). Second, even if feedback loops are essential to adaptive phenomena in photoreceptors, it is conceivable that they are well-approximated by a feedforward process for a range of inputs. In this approximation, one trades mechanistic details for computational simplicity.

One may wonder why phototransduction requires several feedback loops (illustrated in Fig. 9) and which aspect of visual computation they each relate to. One way to approach the problem is to identify a computation that a feedforward system *cannot* carry out. Somewhat surprisingly, we found that a feedforward system, such as the DA model, can reproduce sophisticated data with high accuracy. It is thus plausible that a non-linear feedforward system with several time scales mimics feedback quite well. Alternatively, it is possible (though improbable) that some of the feedback is necessary, not for functional computation, but for internal molecular bookkeeping or as a safety net when photoreceptors face extreme conditions.

Carpenter and Grossberg have proposed several variants of phototransduction models [32]. One among these can be re-written in a way that makes the similarity with the DA model apparent. The important difference between the two, though, is that the Carpenter and Grossberg model is devoid of a delayed process, as opposed to the DA model which captures delayed effects with its 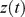 term.

Away from the specific realm of phototransduction, a number of studies of cellular signal transduction have introduced models that share similarities with the DA model; see Refs. [33]–[38] and references therein. In most cases, however, the decay part of the model equation contains only a non-linear term, while in the DA model it has both linear and non-linear components. One such example is referred to as the ‘perfectly adapting’ model in the cell signaling literature. One model of phosphorylation-dephosphorylation contains both linear and non-linear decay. But it is governed by a single time scale in the signal. By contrast, the interplay of different time scales, which appear through the 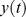 and 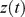 terms in the DA model, is central to its behavior. To our knowledge, the present work provides a novel application of dynamical systems ideas popular in signaling studies to transduction cascades in neurons, and offers detailed results on adaptation to stimuli with complicated correlation structures.

### How Widely Are DA-Like Models Valid?

We applied the DA model to turtle cone and salamander cone data, but we anticipate that it can be used to describe photoreceptors in other taxa since these exhibit a very similar phenomenology (Table 1). The trends we discussed in the context of experiments on the turtle cone are consistent across species, from invertebrates such as fly and Limulus to vertebrates such as salamander, mouse, and primate. In particular, modulation of both gain and dynamics is observed across taxa. Every studied species exhibits non-linear compression of the flash response in the dark as well as speed-up with flash intensity. Gain compression as a function of background light intensity is also apparent in all species, although in some cases it is best fitted with a modified, non-linear Weber-Fechner rule (according to which the response is proportional to 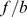, the ratio of the flash intensity to the background intensity, raised to some power).

**Figure 10:**
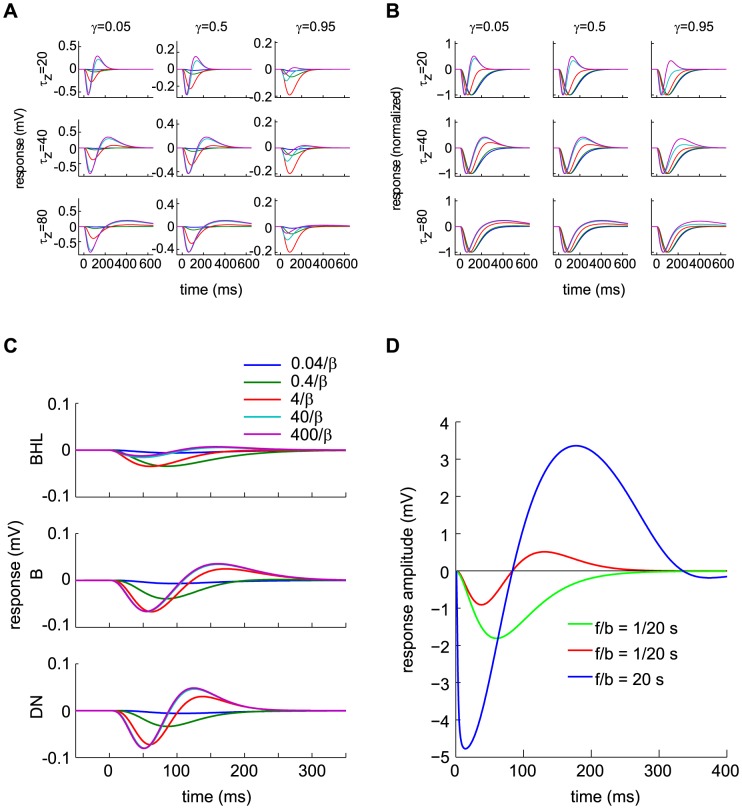
Behavior of the DA model for different parameter values. (A) and (B) Families of responses to a flash in different light backgrounds. We use the BHL parameter set as default parameter set, with changes in 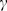 and 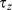 as indicated. The flash intensities take the values 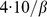, 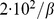, 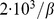, 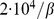, 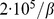, with the background light intensities ranging from 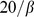 to 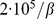 by factors of 10, respectively. (A) and (B) represent identical curves; traces were normalized by their peak values in (B) so that shapes can be compared. As 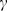 increases from 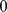 to 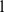, the shape of the saturated curves remain unchanged, but the onset of the non-linearity and the amplitude of the curve are affected. As 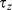 increases, the second, overshoot lobe becomes shallow, and hence more difficult to observe (especially in the potential presence of noise). (C) Comparison of the three sets of model parameters used to fit data. Responses to a flash superimposed upon a light background are displayed for different background intensities. In each panel, the five curves correspond to background intensities increasing from from 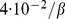 to 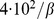 by factors of 10; the associated flashes occur at time 0, last for 1 ms, and have unit Weber contrast, i.e., have equal intensity to that of the background. We note that the value of gamma in the BHL panel is higher than in other panels likely because it was fit only to flash responses in the dark, so that amplitude shifts at high background were not included in the fitted data. (D) DA model responses to a weak flash against a dim background (green, 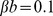), a weak flash against a bright background (red, 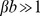), and an intense flash against a bright background (blue, 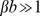). Note that, in the presence of a bright background, zero crossings always occur at the same point and, despite the 400-fold difference in flash strength, the intense-flash response is only 5-fold greater than the weak-flash response.

Biphasic (‘differentiating’) flash responses in the presence of a bright background are observed widely, and indeed the DA model predicts a transition from monophasic impulse responses to biphasic impulse responses for increasing background intensity. Yet, it appears that the second, overshoot lobe may be less pronounced or even absent in some species, such as primates, as compared to the turtle data examined in detail here. Also, while much of the insect data does not present biphasic flash responses in bright backgrounds, the literature cited in Table 1 notes a small but distinct second lobe. (Interestingly, however, laminar recordings in insects display perfect biphasic impulse responses that integrate to zero [60].) The DA model can account for such variations in the shape of the impulse response. In particular, the parameter 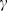 sets the background intensity at which an overshoot lobe appears and the parameter 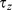 sets the shallowness of the overshoot lobe; for large 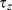, the overshoot becomes very shallow and can be difficult to detect in the presence of noise. (See Fig. 10A and B and captions for a more detailed discussion of this point.)

Since invertebrate and vertebrate phototransduction cascades are evolutionarily distinct [61], one is led to think that the adaptation phenomenology summarized in Table 1 represents an adequate solution to the problem of encoding natural visual inputs [4]. Downstream visual neurons [1], [2], [5]–[8], [10], [62]–[67] and, indeed, neurons in the other sensory systems [68] display adaptive properties similar to those recorded in photoreceptors. A model in the spirit of the DA model may be suitable for these. What refinements or elaborations of the DA model would then be required—more complicated temporal filters? a broader range of time scales? a more involved form of the non-linearity? several non-linear stages?—is itself an interesting question.

## Methods

### Fitting the DA Model to Data

Since the pioneering work of Hodgkin and Baylor [13], [69], standard functional forms have been used to fit the impulse response of visual neurons, and we found that these forms indeed appropriately fit all the data we examined. By convention, we require that the filters 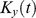 and 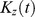 each integrate to unity. For 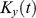, we adopted the form
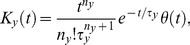
(12)where 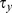 specifies the time scale of the linear response, 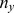 specifies its ‘rise’ behavior, and 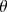 is the Heavyside function with 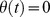 if 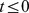 and 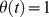 if 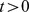. This filter corresponds to a sequence of 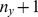 simple relaxation equations in time, as may occur in the phototransduction cascade. While other, more involved choices may yield a closer quantitative agreement with data, we found that a similar form for 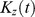, with the added twist that it combines two time scales, is satisfactory. Specifically, throughout we used the form
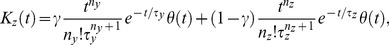
(13)according to which 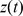 involves a fast component that responds on the time scales of the linear response, 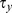, and a slow component that responds on a somewhat longer time scale, 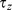. The prefactors, 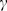 and 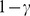, weigh the relative importance of the two components and ensure normalization to unity.

Throughout, we integrated the DA model with standard techniques in Matlab (Mathworks, Natick, MA). We fit our model parameters separately to each of the three data sets we used, from the experiments of Baylor, Hodgkin, and Lamb [13]–[15], the experiments of Burkhardt [18], and the experiments of Daly and Normann [16]. We used a gradient descent method in Matlab to find the parameter sets that yielded the least squared error from the experimental results. Fits performed with different initial conditions yielded similar parameter minima. In the case of the Baylor, Hodgkin, and Lamb data, the fit was performed to the voltage traces in Figure 2A (extracted from Ref. [14]). All eight parameters of the model, 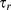, 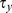, 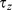, 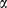, 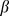, 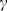, 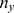, and 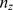, were varied in the minimization procedure. For the Burkhardt data, the optimized parameters were determined from the family of curves in Fig. 5D (extracted from Ref. [18]). The parameters 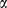, 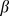, 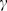 were varied; because these data represented not traces but amplitudes, the remaining parameters had little effect on the fit and were set to typical values before the fitting routine was applied. For the Daly and Normann data, the fit was performed on the flash response traces in Fig. 5A (extracted from Ref. [16]). All eight parameters of the model, 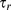, 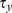, 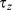, 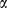, 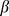, 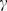, 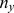, and 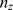, were again varied to find the least squared error between the DA model responses and experimental flash responses. The value of 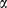 was adjusted from experiment to experiment within the same data source, to match the scale. Nonetheless, 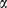 remained in the vicinity of 2 mV⋅µm^2^⋅ms/photon (see Table 2), where we have assumed a cone cross-section of 1 µm^2^. This value of 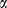 yields a peak dark hyperpolarization of ∼15 µV/photon in agreement with experimental observations. The optimized parameter sets are recorded in Table 2. For Fig. 2F, where the experimental flash intensity was unspecified, the flash intensity, rather than 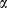, was adjusted so as to obtain response strengths comparable to the data. Figure 10C displays the flash response for different values of the background light intensity, for each of the three parameter sets used in fitting the data. Again, we emphasize that the results are robust with respect to parameter changes and fits by eye resulted in similar parameters and goodness of fit. The values of 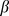 and 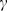 determine the location of the crossover to a non-linear behavior, as well as the relative strength of the effect, but have relatively little effect upon the shape of saturated flash responses (see Fig. 10A and B).

For Fig. 8, the light intensity time series was extracted from van Hateren's recordings of naturalistic stimuli [50] and the goldfish cone response was digitized from Ref. [49]. In calculating the DA model output, the parameters 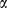, 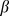, 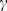 were fit to the goldfish cone traces for Fig. 8A, where we used a mean light intensity of 1.5·10^5^  photons/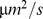 (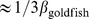) [49]; the BHL time scales were used in the model as the low temporal resolution of the digitized trace did not allow for a more precise temporal fit. For Figs. 8B, C, and D, parameter set B was used together with a mean light intensity of 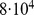 photons/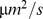 (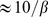). The gain was probed by superimposing 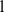 ms flashes containing 100 photons on top of this fluctuating light background. Flash responses were found by subtracting the response to the naturalistic time series from the response to the same time series with superimposed light flashes.

### Salamander Experiments and Analyses with LN and DA Models

Salamander retinæ were exposed to whole-field flicker of time-varying intensity while a sharp electrode voltage recording was made of cone cells, following the protocol of Ref. [8]. Flicker was presented with a CRT at 67 Hz, with a mean light intensity of 
∼ 10mW/m^2^. Intensities were updated every 2 frames and chosen from a Gaussian distribution with standard deviation equal to 35% of the mean. All data integration and analysis were performed with custom-written routines in Matlab. (We provide some of these codes as online supplementary material.)

*LN model analysis*. Best-fit linear filters were found using cross-correlation methods, as described in Ref. [8]. A 500 ms filter was used to produce the linear portion of the LN model output, followed by a third degree polynomial fit of that output to the experimental response. In order to compute the quantities in Figs. 6G–I, windows of 300 ms were selected every 100 ms during stimulus presentation. We computed the mean light intensity over each window as well as the ‘instantaneous gain’. The instantaneous gain at a given time was defined as the slope of the scatter of the experimental response when it was plotted against the linearly filtered signal, with scatter points extracted from the trace over the 150 ms windows flanking the time in question (Fig. 6G). We averaged the instantaneous gain over each of the 300 ms time windows to obtain an effective gain associated with a time window as a whole (Fig. 6H). These 300 ms (zero-padded) windows were used to find the peak cross-correlation times of stimulus with response (Fig. 6H) and the linear filters displayed in Fig. 6I. Peak correlation time, for each average of cross-correlations, was defined as the average of the 10 times of maximum correlation. In Fig. 6I, we selected the time points with lowest, middle, and highest instantaneous gains, and plotted the filters corresponding to each of the three subsets of data.

*DA model analysis*. Model parameters were fit to the salamander data (Fig. 6B) with a least squares minimization routine in Matlab and Eq. (2) was integrated with the use of standard methods in Matlab, with the functional forms described above. All parameters (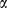, 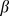, 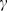, 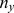, 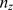, 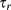, 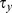, and 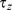) were varied, but 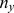 and 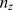 were imposed to take integer values. The parameters used to fit salamander data are recorded in Table 2.

*Analysis of statistical significance*. To assess the significance of the difference in slopes, we applied a Monte Carlo shuffle analysis, in which we ran the slope fitting routine 3000 times, each time offsetting the stimulus and response by a random temporal delay, using circular boundary conditions. The 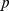-value was calculated as the frequency with which a difference in slopes occurred with absolute value greater than the one measured in the absence of temporal shift. This same random shuffle method was used to assess the timing differences measured in Fig. 6I, using a random shift in the instantaneous gain value, so as to randomly select stimulus-response snippets. These snippets were used to estimate the peak cross-correlation (as in Fig. 6H), and establish an estimated *p*-value for the difference.

### General Analytical Solution of the DA Model

The DA model (defined by Eqs. (2, 3, 4) above) is solved *exactly* for *any* input, by
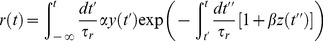
(14) ((Eq. (7) above), where 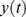 and 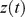 are defined in Eqs. (3, 4). In the case of deterministic inputs such as flashes or steps, this expression can be evaluated readily analytically or numerically. In the case of random inputs, we evaluate this expression for a given instantiation of the noise and then take an average over instantiations. (It is possible to calculate higher moment, such as the variance of _, or the distribution of the response as a whole, in a similar manner, but we do not present the corresponding calculations here.

In some instances below, we compute model photoreceptor responses with the simplifying assumption of small 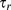. In this limit, the DA model becomes an algebraic equation which can be solved immediately:
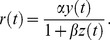
(15)
Results derived in this limit hold also in the case of bright backgrounds.

In order to establish the notation, we now write down the most general input we shall consider here. It is made up of a constant light background, 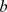, a fluctuating (random) background, 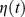, and a flash of intensity 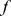 presented at 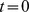:
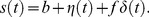
(16)We assume that
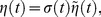
(17)where 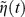 is a Gaussian random variable with temporal correlation
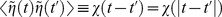
(18)normalized such that
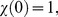
(19)and 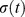 is a deterministic envelope. We shall consider three different cases for the deterministic envelope:

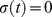
 (no flickering background),

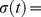
 constant (flickering background with fixed variance),

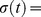
 periodic wave (flickering background with alternating variance).

Note that, with the stimulus in Eq. (16), the filtered quantities read
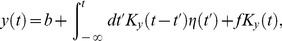
(20)

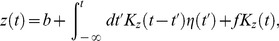
(21)where the kernels 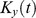 and 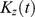 are defined in Eqs. (12, 13) above.

### Solutions of the DA Model for Deterministic Inputs

#### Response to a small flash against a general light background

We calculate the response to a small flash of light, perturbatively in the intensity of the flash. To lowest order, the reduced equation for the response to a small flash, defined as
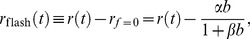
(22)becomes
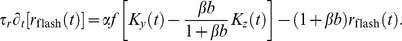
(23)Thus, the response to a small flash of intensity 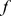 (which measures the total number of photons absorbed by the model photoreceptor) against a background of intensity 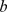 (which measures the number of photons absorbed per unit time) is simply a low-passed version of the pulse
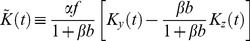
(24)over a time scale
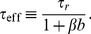
(25)


In dim backgrounds (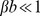), the response is monophasic and close to
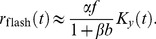
(26)In bright backgrounds (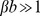), the response becomes biphasic and converges to the perfect biphasic pulse,
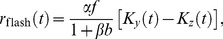
(27)which integrates to zero, in very bright backgrounds. Thus, in dim backgrounds the photoreceptor integrates incoming photons over the time scale of 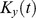, while in bright backgrounds the photoreceptor behaves as a differentiator over the time scales of 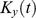 and 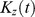. (Note that the delay 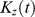 relative to 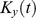 is paramount to this behavior.) Equivalently, a dark-adapted model photoreceptor is low-passing while a light-adapted model photoreceptor is band-passing.

Figure 1A illustrates the kernels 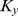 and 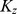 and Fig. 10D exhibits flash responses in dim and bright light-adapted conditions.

#### Response to a general flash against a bright background

In bright backgrounds (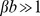), the effective time scale, 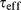, becomes negligible and, consequently, the DA model reduces to the simpler algebraic form
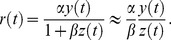
(28)The flash response (as defined above in Eq. (22)) then becomes
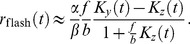
(29)There are several qualitative statements one can extract from this expression. First, in bright backgrounds the response depends only upon the ratio 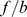 (not upon 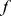 and 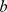 independently). Second, the time of the node of the biphasic response (given by 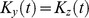) does not change with either 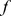 or 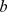. Third, for large flashes the response reduces to a fixed profile,
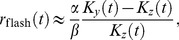
(30)over the time window in which 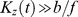. (See Fig. 10D.)

### Solutions of the DA Model for Randomly Fluctuating Inputs

The general solution of the DA model, Eq. (7), can be rewriten as
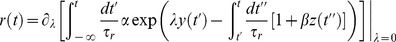
(31)or as
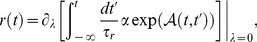
(32)with
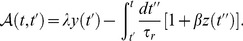
(33)


The average response, over instantiations of the flicker, is then given by
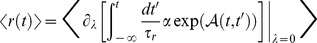
(34)

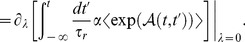
(35)Since all random variables in the problem are linear sums of Gaussian variables, we have
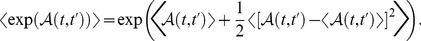
(36)After replacing the variables 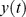 and 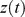 by their expressions in terms of inputs and filters, Eqs. (20, 21), and performing the Gaussian averages, we obtain the average response
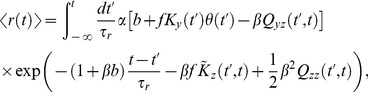
(37)where
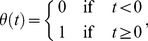
(38)

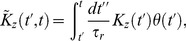
(39)

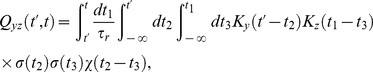
(40)

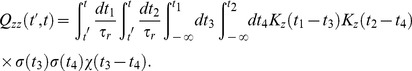
(41)This average response includes a tonic component, the baseline response to the constant background, and a phasic component, the flash response. Because of the non-linearity in the DA model, both components are modulated by the flicker, as compared to the deterministic case.

Hereafter, we examine this solution in two cases: flicker with constant variance and flicker with periodically varying variance. We answer the following question: How does flicker affect the phasic and tonic components of the model photoreceptor response?

#### Modulations of the mean response and flash response by flicker with constant variance

**Figure 11:**
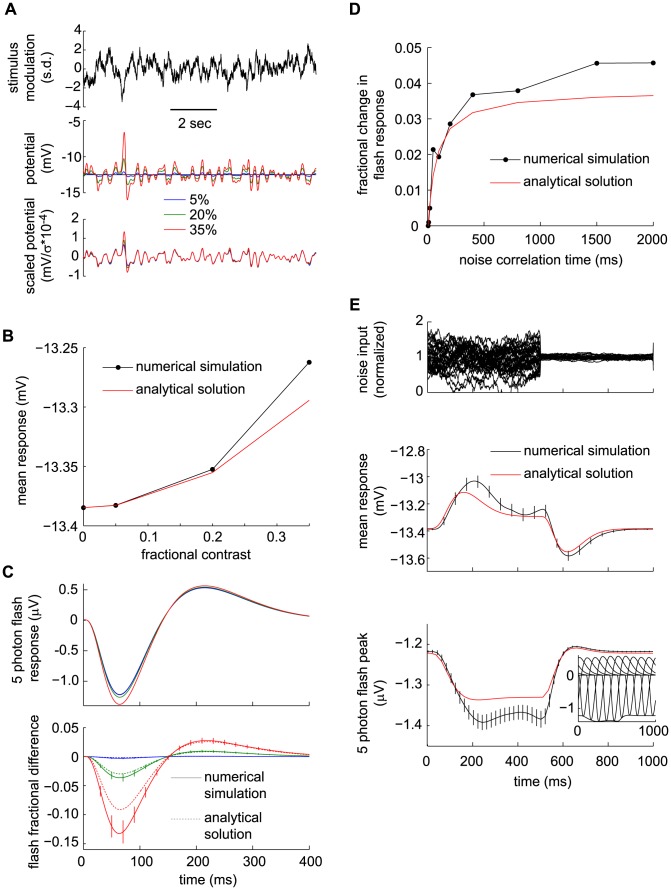
Behavior of DA model responses in the presence of Gaussian fluctuating inputs. All calculations in this figure use parameter set B. (A) Top: A sample Gaussian input with correlation time of 200 ms. Middle: Model responses for three different contrasts of the fluctuating input. The mean input intensity is given by 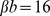. Bottom: After normalization of the response by the input's standard deviation, one can see signatures of the model's non-linearity as the curves do *not* collapse unto a single curve. (B) Mean response of a model photoreceptor presented with Gaussian flickers with three different contrasts: analytical and numerical results. Larger contrasts yield less hyperpolarized responses, on average. Black dots: numerical result; red curve: analytical result. (C) Mean flash responses of a model photoreceptor in the presence of Gaussian flickering backgrounds with different variances. Top: Average flash responses were calculated numerically by running simulations with two different stimuli: one with Gaussian flicker only, the other with Gaussian flicker and superimposed flashes. The average flash response was obtained as the difference between the two outcomes, averaged over flicker instantiations. Bottom: The fractional difference between flash responses in the presence of Gaussian flicker with three different contrasts. Solid lines: numerical result (±1 SEM error bars); dotted lines: analytical result. (D) Fractional change in average flash response as a function of flicker correlation time. From Eqs. (44, 45, 46), the magnitude of the average flash response depends upon the correlation time of the random flicker. Black dots: numerical result; red curve: analytical result. (E) Responses of a model photoreceptor in the presence of flicker with time-varying contrast. We fed the DA model Gaussian flicker with standard deviation alternating between 35% and 5%, with a 1 second period (top). Average flash responses were calculated at different times during the period (middle), as was done in (C). Sample average flash responses are displayed in the inset panel, while the main panel shows the variation of the flash response amplitude across one period. Black curve: numerical result (±1 SEM error bars); red curve: analytical result. The average response (bottom) was also calculated numerically (black, ±1 SEM) and analytically (red). Note the overshoots of the average response following contrast switches (see the text for an explanation).

For time-independent flicker, we have
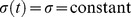
(42)(Fig. 11A).

The phasic response (*i. e.*, the average flash response in the presence of flicker) is defined as
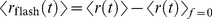
(43)and can be extracted from Eq. (37) similarly to 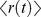, above. For the sake of simplicity, we consider the limit of a bright background and small flash and flicker standard deviations. Our calculations are carried out to lowest non-trivial orders in 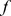 and 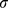. Expanding Eq. (37) is these limits, we obtain
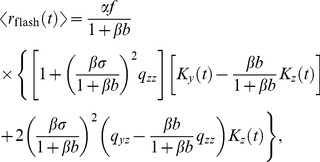
(44)where
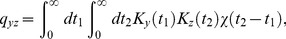
(45)

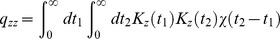
(46)(Fig. 11C). These quantities measure an ‘effective overlap’ of the linear filters, weighed by the temporal correlation function of the flicker. If the flicker is correlated over short time scales, then 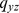 and 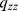 are true overlaps of the filters and, since 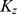 is delayed with respect to 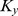, 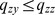. If the flicker is correlated over long time scales, then the integrals decouple and 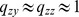. As compared to the flicker-free case, the flash response is boosted by a multiplicative factor
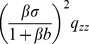
(47)and it acquires an additive contribution equal to
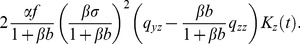
(48)We note that the multiplicative term is always positive, and hence corresponds to a gain enhancement due to flicker. Also, since the quantities 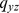 and 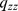 depend upon the temporal correlation of the flicker, so will the flash response (Fig. 11D). In other words, the model photoreceptor adapts to both the magnitude and the temporal structure of the input.

**Figure 12:**
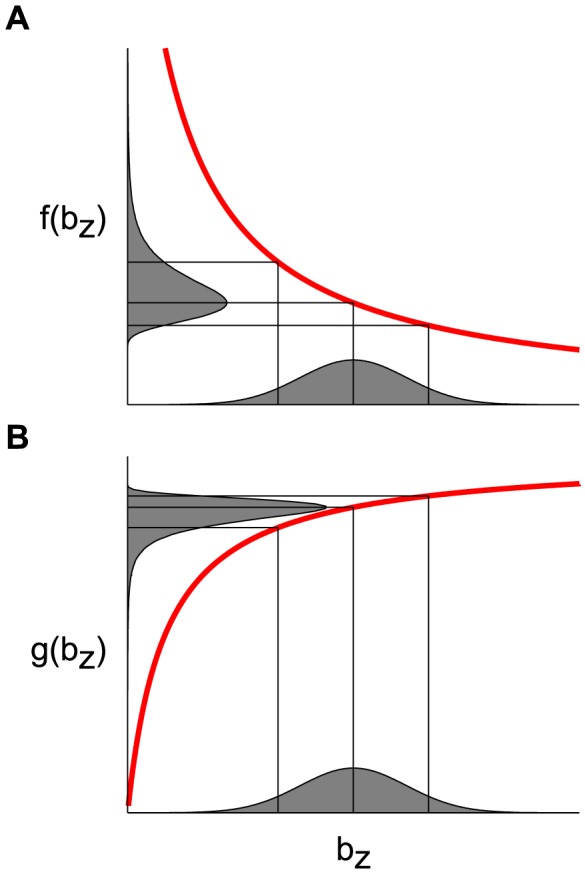
Illustration of the non-linear transformation of fluctuating inputs. (A) A concave functional form, as that of the gain prefactor in the flash response (Eq. (49)), increases the mean in the transformation. That is, downward fluctuations in 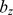 contribute more to increasing 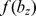 than upward fluctuations to decreasing it. Therefore, 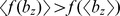. The flash response gain in the presence of a fluctuating background is thus larger than that in the presence of a constant, mean-matched background. (B) A convex functional form decreases the mean in the transformation, so that 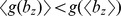. Depending upon the strength of the background, 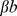, and the relative structure of the two kernels, 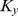 and 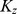, the mean response to a fluctuating input may be either suppressed or enhanced compared to the flicker-free case. The DA model (see, e. g., Eq. (55)) takes on the convex form shown here as 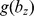 in the case with 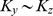 (and 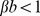), thus decreasing the mean response relative to that in the presence of a constant input. In the case in which 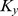 and 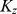 differ in their timing, the functional form becomes concave as in (A) and the mean response to a fluctuating input is enhanced. The modulation of the mean response therefore depends sensitively upon stimulus and kernels parameters.

It is instructive to consider the limit of small 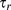 in order to intuit this result. In that limit, the (unaveraged) flash response reads
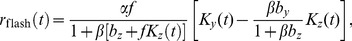
(49)where we have introduced the quantities
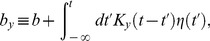
(50)

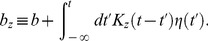
(51)These are fluctuating backgrounds that modulate the overall gain and the magnitude of the overshoot. The overall gain prefactor, 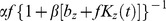, is a concave function of 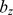. As a result, when 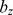 fluctuates about its mean value, 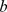, the average gain is enhanced as compared to the flicker-free case with 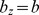 (Fig. 12A). The modulation of the overshoot behaves in a subtler manner, because it involves both 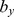 and 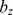. Since the same term governs the behavior of the mean (tonic) response of the model receptor, we return to this discussion below (see also Fig. 12B).

But before treating the mean response, we mention that a generalized version of the result in Eq. (44) which includes the full non-linear contribution of the flash intensity may be derived from Eq. (49). To the first non-trivial order in the flicker, we obtain
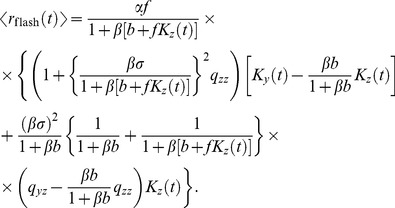
(52)This result corroborates that in Eq. (44) and extends it to cases of large flash intensity.

The tonic response (*i. e.*, the mean response to constant background plus flicker) is defined as
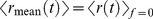
(53)and can also be extracted from Eq. (37). In the presence of flicker, it acquires a flicker-dependent contribution and is calculated as
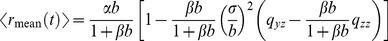
(54)(Fig. 11B). Depending upon the intensity of the background, 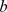, the temporal structure of the kernels, and the relative magnitude of the fast and slow components in the kernel 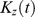 (prescribed by the parameter 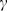), the flicker-dependent contribution may suppress or enhance the mean response.

In order to intuit this result, it is again useful to study the limit of small 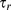, in which the (unaveraged) flicker-dependent part of the response is expressed as
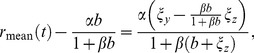
(55)where we have introduced the quantities
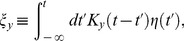
(56)

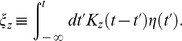
(57)(Fig. 11A). One can think of the mean response as a cumulative response to a succession of many bright and dark flashes with random intensities. The usual, linear, bi-lobe response, 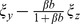, is modulated by the denominator, 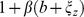. It is easy to understand the behavior of 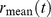 by sketching the form of the bi-lobe response together with the form of the non-linear modulation. An important point is that this modulation is asymmetric for bright and dark flashes. If the background intensity, 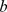, is low or if the kernel 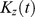 is not too different from the kernel 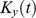, then, on average, the mean response is *suppressed* compared to the flicker-free case (see Fig. 12B). If the background intensity, 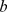, is high or if the kernel 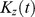 is significantly delayed with respect to the kernel 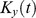, then, on average, the mean response is *enhanced* compared to the flicker-free case (see Fig. 12).

#### Modulations of the mean response and flash response by flicker with time-varying variance

Here, the flicker amplitude, 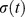, depends upon time. For now, we assume a general function; below, we focus on the special case of a periodic function (Fig. 11E). The analytical treatment is very similar to the case of constant-variance flicker, so here we confine ourselves to quoting the main results.

The phasic response (*i. e.*, the average flash response in the presence of flicker) reads
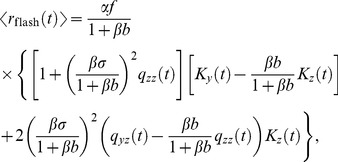
(58)where
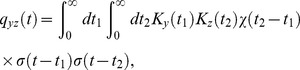
(59)

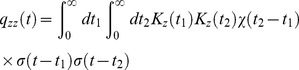
(60)(Fig. 11E). Equation (58) reduces to Eq. (44) if the standard deviation of flicker is constant. Similarly to the constant-variance case, Eq. (58) implies that the flash response increases by a time-dependent multiplicative factor
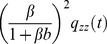
(61)and acquires a time-dependent additive contribution equal to
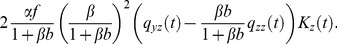
(62)


The tonic response (*i. e.*, the mean response to constant background plus flicker) becomes
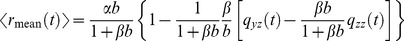
(63)(Fig. 11E). Equation (63) reduces to Eq. (54) if the standard deviation of flicker is constant. For large background intensity, 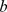, Eq. (63) becomes
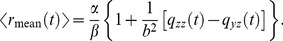
(64)


In Results, we consider the particular case of a periodic, square-wave envelope of the flicker, with standard derivation that switches between a low value, 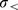, and a high value, 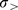. Equation (64) implies that, when 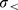 switches to 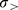, the tonic response undergoes an overshoot; this is because the peak of 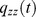 is delayed with respect to that of 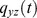.

## Supporting Information

File S1 **DA_model_script.txt.** This document is a Matlab script designed to run with File S2. It demonstrates some of the features of the DA model, and provides an implementation of the model. Informative comments are included in the code.
(TXT)

File S2 **DA_integrate.txt.** This document implements the dynamical adaptation model in Matlab, using structures defined in File S1. PLoS Computational Biology does not recognize *.m files for supplemental material; in order to work, users must change the ‘txt’ suffix, in both file names, to ‘m’.
(TXT)
